# AURKA regulates LGR5 in response to *Helicobacter Pylori* infection by modulating its deubiquitination

**DOI:** 10.1038/s41418-026-01810-w

**Published:** 2026-07-11

**Authors:** Ahmed Gomaa, Selma Maacha, Mohammed Soutto, Silvia Giordano, Nadeem Bhat, Shria Kumar, Oliver G. McDonald, Wael El-Rifai

**Affiliations:** 1 Department of Surgery, University of Miami Miller School of Medicine, Miami, FL, USA.; 2 Department of Veterans Affairs, Miami Healthcare System, Miami, FL, USA.; 3 Department of Oncology, University of Torino and Candiolo Cancer Institute, Candiolo, Italy.; 4 Department of Medicine, University of Miami Miller School of Medicine, Miami, FL, USA.; 5 Sylvester Comprehensive Cancer Center, University of Miami, Miami, FL, USA.; 6 Department of Pathology and Laboratory Medicine, University of Miami Miller School of Medicine, Miami, FL, USA.

## Abstract

Aurora kinase A (AURKA) is frequently overexpressed in gastrointestinal cancers. *Helicobacter pylori (H. pylori*) infection is a significant risk factor for gastric carcinogenesis. LGR5, a stem cell marker in the stomach, plays an important role in gastric tumorigenesis. This study investigates the link between AURKA and LGR5 in response to *H. pylori* infection in gastric cancer. We analyzed publicly available datasets, gastric cancer cell lines, patient-derived organoids and xenografts (PDOs and PDXs), mouse models, and de-identified human tissue samples. We found a strong association between *AURKA* and *LGR5* expression levels in gastric cancer tissues. In vitro and in vivo analyses showed increased AURKA and LGR5 protein levels in response to *H. pylori* infection. Using cell models and PDOs, we demontrated an AURKA-dependent induction of LGR5, whereas genetic knockdown or pharmacologic inhibition of AURKA abolished the *H. pylori*-induced increase in LGR5 by enhancing LGR5 ubiquitination. Mechanistically, AURKA interacted with STAMBP, suppressing LGR5 deubiquitination and thereby promoting its stabilization. Using a tamoxifen-induced conditional knockout (CKO) mouse model of *Aurka* (*Krt19^CreErt^/Aurka*^flox/flox^), we detected a lower baseline expression of LGR5 in gastric glands, with reduced induction following *H. pylori* infection, compared to controls. The *Aurka* CKO mouse model was crossed with the *Tff1*^−/−^ model of gastric tumorigenesis to create the *Krt19^CreErt^/Aurka*^flox/flox^ /*Tff1*^−/−^ mouse model. AURKA knockout, after tamoxifen treatment, significantly reduced the LGR5 protein level and led to a marked decrease in antral thickness. In PDX models, the use of a combination treatment of AMG900 (a pan-AURORA inhibitor) with docetaxel was more effective than monotherapy, lowering LGR5 levels and suppressing tumor growth. In summary, there is a functional link between AURKA and LGR5, mediated by STAMBP to promote stem-like properties and enhance cell survival in gastric tumorigenesis. Targeting the AURKA-LGR5 axis is a potential therapeutic strategy.

## BACKGROUND

Gastric cancer is one of the most prevalent types of cancer, ranking as the fifth most frequently diagnosed malignancy worldwide in 2022 [1]. The majority of patients in the United States are diagnosed at advanced stages (stage III or IV), often with distant metastasis [2]. Gastric cancer is an aggressive malignancy with a poor clinical outcome due to resistance to current therapeutic modalities [1, 3]. A significant factor contributing to this clinical challenge is the presence of cancer cells enriched with stemness properties, a subpopulation of tumor cells with self-renewal capabilities, and resistance to conventional therapies [4].

*Helicobacter pylori (H. pylori)* is a gram-negative bacterium that specifically colonizes the gastric epithelium and represents the most prevalent chronic bacterial infection worldwide. Infection with *H. pylori* results in chronic gastritis, which often persists for decades, establishing a dynamic and enduring equilibrium between the bacterium and its human host. However, this prolonged host-pathogen interaction imposes a significant biological burden [5]. *H. pylori* infection activates oncogenes and tumor suppressor genes through genetic and epigenetic mechanisms, ultimately driving the development and progression of gastric cancer [5–7]. One way in which *H. pylori* drives gastric carcinogenesis is by inducing the expression of Leucine-rich repeat-containing G-protein coupled receptor 5 (LGR5) [8–10]. LGR5 is a well-established stem cell marker in the gastrointestinal tract, including the stomach. LGR5 has been implicated in both normal gastric epithelial homeostasis and cancer progression [11, 12]. LGR5-positive cells contribute to tumor growth, therapeutic resistance, and relapse [13]. Understanding the regulation of LGR5 in gastric cancer pathogenesis is critical for developing effective therapeutic strategies aimed at eradicating cancer stem cells and improving patient outcomes.

Aurora Kinase A (AURKA) is a mitotic kinase enzyme that facilitates centrosome maturation and mitotic spindle assembly under normal physiological conditions [14]. Several studies have demonstrated amplification and / or overexpression of *AURKA* in gastric cancer [15–17]. The aberrant overexpression of AURKA in cancer cells drives several non-mitotic functions through the activation of oncogenic signaling pathways such as NF-κB, β-catenin, STAT3 and EIF4E [14, 16, 18, 19]. However, its role in regulating stem-like properties has not yet been fully understood.

The ubiquitin-proteasome system (UPS) is a key protein quality control pathway found in all eukaryotic cells. Ubiquitination is a highly regulated process that occurs through a series of steps involving three main enzymes: the E1 activating enzyme, the E2 conjugating enzyme, and the E3 ligase [20]. The UPS plays a crucial role in regulating numerous essential biological processes by affecting the stability, activity, and subcellular localization of target proteins [21]. Normally, ubiquitination can be reversed by deubiquitinases (DUBs), which remove ubiquitin from target proteins, preventing their degradation by the proteasome and promoting their stability. However, under pathological conditions such as cancer, impaired DUB function can disrupt the ubiquitin-proteasome system (UPS), leading to increased protein stability and accumulation [22]. Recent studies indicate that dysregulation of DUBs may play a key role in the pathogenesis of gastric cancer [23]. The DUB enzyme STAMBP is known to be overexpressed and amplified in multiple types of cancer [24–26]. The functions of STAMBP in gastric cancer remain unclear. In this study, we report that AURKA regulates LGR5 protein levels by modulating its deubiquitination through increased STAMBP activity.

## RESULTS

### AURKA promotes organoids’ growth and increases LGR5 levels

To investigate whether deletion of AURKA affects mouse gastric organoid growth, gastric organoids from wild-type mice and *Krt19*^CreErt^/*Aurka*^flox/flox^ mice [15], in which *Aurka* deletion occurs specifically in *Krt19*-expressing gastric epithelial cells, were treated with 0.5 μM tamoxifen for 24 h in vitro to induce *Aurka* deletion. Organoids derived from wild-type mice exhibited no changes in growth, whereas organoids derived from *Krt19*^CreErt^/*Aurka*^flox/flox^ mice exhibited a significant reduction in size (Fig. 1A, B). Next, we overexpressed AURKA in gastric organoids derived from normal human gastric tissue. These organoids were transduced with pAV[Exp]-EGFP-CMV>h*AURKA* adenovirus at a multiplicity of infection (MOI) of 25. As a result, AURKA-overexpressing organoids showed a significant increase in size (at day 8) compared with those transduced with the empty vector control (Fig. 1C, D). To uncover the key molecular functions associated with AURKA that might contribute to organoid growth, we analyzed the TCGA-STAD dataset alongside seven publicly available GEO datasets (GSE122401, GSE54129, GSE66229, GSE15459, GSE110875, GSE26899, GSE26901). This analysis revealed that processes related to stem cell differentiation, cell division, and proliferation, as well as *β-catenin* signaling, were among the most significant enriched molecular functions linked to *AURKA* overexpression (Fig. 1E, F). Notably, *AURKA* expression was significantly correlated with *LGR5*, a known gastric stem cell marker, in multiple gastric cancer GEO datasets (Fig. 1G). To assess whether *Aurka* deletion affects LGR5 expression, cells were isolated from gastric organoids derived from both wild-type (WT) and *Krt19*^CreErt^/*Aurka*^flox/flox^ mice [15]. As shown in Fig. 1H, deletion of *Aurka* led to a significant reduction in LGR5 protein levels in *Krt19*^CreErt^/*Aurka*^flox/flox^ organoids, suggesting a potential regulatory role for *Aurka* in modulating *Lgr5* expression in mouse gastric organoids. Using human gastric cancer cell models, *AURKA* overexpression via a doxycycline-inducible Tet-On system resulted in increased LGR5 protein levels after 48 h of doxycycline treatment, with LGR5 expression returning to baseline upon doxycycline withdrawal (Fig. 1I). Similarly, overexpression of AURKA using pAV[Exp]-EGFP-CMV>h*AURKA* adenoviral transduction significantly increased LGR5 protein levels (Fig. 1J). In contrast, *AURKA* knockdown using *AURKA*-specific siRNA significantly decreased LGR5 expression in both AGS and STKM2 gastric cancer cell lines (Fig. 1K, L). Furthermore, pharmacologic inhibition of AURKA with AMG-900 led to a marked reduction in LGR5 protein levels in both AGS and STKM2 cell lines (Supplementary Fig. 1A, B). Collectively, these findings support the presence ofa regulatory role for AURKA in controlling LGR5 level in vitro and in vivo.

### *H. pylori* infection induces AURKA and LGR5 expression

*H. pylori* infection is the main risk factor in gastric carcinogenesis [8]. To examine the impact of *H. pylori* infection on the expression of LGR5 and AURKA in human gastric organoids, we dissociated normal gastric organoids into single cells and infected them with the *H. pylori* strain 7.13 for 3 h. The infected cells were then embedded in Matrigel and cultured at 37°C for one week. *H. pylori* infection significantly increased both the size and number of organoids (Fig. 2A, B). Immunofluorescence analysis of normal human gastric organoids infected with *H. pylori* strain 7.13 demonstrated a significant upregulation and pronounced colocalization of AURKA and LGR5 compared to uninfected controls (Fig. 2C, D). Similarly, wild-type mice infected with the mouse-adapted *H. pylori* strain PMSS1 displayed markedly elevated expression and colocalization of AURKA and LGR5 in the mouse antrum (Fig. 2E, F). These results were confirmed by Western blot and quantitative RT-PCR analyses (Fig. 2G–I). In addition, infection of gastric cancer cell lines AGS and STKM2 with *H. pylori* strain 7.13 resulted in a significantly increased expression of both AURKA and LGR5 (Fig. 2J, K). On the other hand, infection of gastric cancer cell lines (AGS and STKM2) with the CagA-negative *H. pylori* strain 7.13 did not result in any significant changes in the expression of either AURKA or LGR5 (Supplementary Fig. 1C, 1D). These results suggest a functional association between AURKA and LGR5 during *H. pylori* infection.

### AURKA knockdown suppresses *H. pylori*-dependent LGR5 induction

To investigate the effect of AURKA on LGR5 expression following *H. pylori* infection, we used RNA interference to knock down *AURKA* (siAURKA) in AGS and STKM2 cells. The results showed a notable decrease in LGR5 protein levels after *AURKA* knockdown (Fig. 3A, B). To further elucidate the role of AURKA in the *H. pylori*-mediated regulation of LGR5, we utilized the *Krt19^CreErt^/Aurka*^flox/flox^ conditional knockout mouse model [8]. First, we examined the impact of Aurka deletion on organoid growth and size. Single cells were isolated from the gastric glandular epithelium of mice infected with PMSS1, with or without tamoxifen (TAM) treatment. *Aurka* deletion in tamoxifen-treated mice resulted in a significant reduction in organoid size (Supplementary Fig. 2A, 2B). To assess the impact of *Aurka* deletion on LGR5 protein expression following *H. pylori* infection in vivo, we conducted immunofluorescence staining on glandular antral stomach tissues from mice infected with PMSS1. Consistent with the in vitro findings, untreated control *Krt19^CreErt^/Aurka*^flox/flox^ (Ctrl) exhibited baseline expression of AURKA and LGR5, which was significantly reduced upon TAM-induced *Aurka* knockout (Fig. 3C, D). Similarly, *H. pylori*-infected control mice (PMSS1) displayed elevated AURKA protein levels accompanied by strong LGR5 immunostaining, compared with uninfected controls (Ctrl) (Fig. 3C, D). Notably, mice subjected to both TAM treatment and *H. pylori* infection (TAM + PMSS1) demonstrated a marked reduction in LGR5 expression relative to infected controls (PMSS1), suggesting that *Aurka* ablation in gastric epithelial cells significantly attenuates LGR5 protein levels in vivo (Fig. 3E). Quantitative analysis of mRNA levels revealed that *H. pylori*-infected control mice (Ctrl+PMSS1) exhibited a significant upregulation of *Aurka* transcripts compared with uninfected controls (Ctrl). This induction was completely abrogated in TAM-treated and *H. pylori*-infected mice (TAM + PMSS1) (p < 0.01, Fig. 3F, G), further supporting the critical role of *Aurka* in modulating *H. pylori*-dependent *Lgr5* expression in mice. Next, we tested the effect of pharmacological inhibition of AURKA, using AMG-900, on human organoids by immunofluorescence or Western blotting. We found that AMG-900 not only abrogated *H. pylori*-mediated increases in LGR5 protein levels (Fig. 3H–J) but also inhibited organoid growth (Supplementary Fig. 2C, 2D). Collectively, our results demonstrate a critical role of AURKA in regulating LGR5 levels and organoid growth in response to *H. pylori* infection.

### AURKA knockdown suppresses tumorigenesis in the *Tff1*^−/−^ mouse model of gastric cancer

Our previous work demonstrated that *Aurka* is upregulated and promotes chronic inflammation and tumorigenesis in *Tff1*-knockout mice [19]. To examine whether *Aurka* knockout modulates *Lgr5* level in this model, we crossed *Krt19^CreErt^/Aurka*^flox/flox^ mice [15] with *Tff1*^−/−^ mice. The resulting *Krt19^CreErt^/Aurka*^flox/flox^/*Tff1*^−/−^ mice allowed for tamoxifen-inducible and stomach epithelium-specific ablation of *Aurka* through Cre-mediated recombination in *Krt19*-expressing cells (Fig. 4A). We administered tamoxifen to 3-month-old mice and collected gastric tissue when they reached 6 months of age. Notably, *Aurka* CKO following tamoxifen treatment resulted in a significant reduction of antral thickness and inflammation score compared with untreated control mice (Fig. 4B–D). To assess the impact of *Aurka* deletion on organoid-forming capacity, we quantified the number of organoids generated from gastric tissues of tamoxifen-treated mice. Organoids derived from tamoxifen-induced *Aurka* knockout mice exhibited a significant reduction in both their number and size compared to organoids derived from untreated controls (Fig. 4E, F).

### Genetic deletion of *Aurka* reduces the population of LGR5+ cells in gastric tumors

To examine the effect of *Aurka* knockdown on *Lgr5* expression in vivo, we used the *Krt19^CreErt^/Aurka*^flox/flox^/*Tff1*^−/−^ mouse model. We have found that tamoxifen-induced *Aurka* CKO resulted in a marked reduction in LGR5 protein levels in gastric tumors compared to control mice (Figs. 5A, B, and Supplementary Fig. 3A). To validate these findings, gastric organoids were established from *Krt19^CreErt^/Aurka*^flox/flox^ /*Tff1*^−/−^ mice and wild-type (WT) controls and cultured until well-developed organoid structures were formed. Organoids were then treated in vitro with tamoxifen for 24 h. to induce *Aurka* deletion. The tamoxifen treatment (TAM) led to a significant suppression of organoid growth in the *Krt19^CreErt^/Aurka*^flox/flox^ *Tff1*^−/−^ group, while organoids derived from wild-type (WT) mice showed no response to tamoxifen, indicating a critical role for AURKA in sustaining gastric organoid proliferation in this genetic background. In contrast, WT organoids exhibited no detectable changes in growth following tamoxifen treatment (Fig. 5C, D). Consistent with these findings, immunofluorescence and Western blot analysis demonstrated a notable reduction in LGR5 protein levels in *Krt19^CreErt^/Aurka*^flox/flox^ *Tff1*^−/−^ derived organoids compared to the organoids derived from wild-type controls (Fig. 5E, Supplementary Fig. 3B, 3C).

Flow cytometric analysis of LGR5-positive cells in gastric tissue from *Krt19^CreErt^/Aurka*^flox/flox^ *Tff1*^−/−^ mice revealed a significant increase in LGR5-positive cells compared to wild-type (WT) mice. Interestingly, gastric tissue from tamoxifen-treated *Krt19^CreErt^/Aurka*^flox/flox^ *Tff1*^−/−^ mice showed a significant decrease in LGR5-positive cells upon *Aurka* deletion (Fig. 5F, G). To assess the function capacity of LGR5-positive cells, organoid assays were performed using sorted LGR5+ cells from the antrum of both tamoxifen treated and untreated *Krt19^CreErt^/Aurka*^flox/flox^ *Tff1*^−/−^ mice. Organoid derived from tamoxifen-treated mice (TAM)-LGR5+ cells showed a substantial decrease in the number of organoids, compared with the LGR5+ cells derived from untreated controls (Ctrl) (Fig. 5H, I). This data highly suggests that AURKA regulates tumor growth in the *Tff1* knockout mice by modulating the expression and function of LGR5. Next, we investigated the impact of *Aurka* CKO on gastric tumorigenesis in vivo. Tamoxifen-treated mice with CKO of *Aurka* exhibited a significant decrease in the number of gastric carcinomas (2 out of 8 mice) compared to untreated controls, which displayed a higher incidence of carcinoma lesions (6 out of 9 mice) (Fig. 5I– L).

### AURKA regulates the stability of LGR5 by modulating its ubiquitination

Since *AURKA* knockdown did not significantly affect *LGR5* mRNA levels in STKM2 cells (Supplementary Fig. 4A, 4B), we hypothesized that AURKA plays a critical role in the post-transcriptional regulation of LGR5, possibly at the protein level. To test this hypothesis, we conducted a cycloheximide (CHX) chase assay in AGS cells with and without *AURKA* silencing by RNA interference. We found that LGR5 protein degradation was markedly accelerated in AURKA-knockdown cells compared to control cells (Fig. 6A, B). Given that ubiquitination is a post-translational modification that targets proteins for proteasomal degradation, we investigated whether AURKA regulates LGR5 stability by modulating its ubiquitination. To address this, we silenced *AURKA* using RNA interference and examined the ubiquitination status of LGR5 by immunoprecipitating LGR5 from cells treated with the proteasome inhibitor MG132. Our results demonstrated that LGR5 was efficiently immunoprecipitated using an anti-LGR5 antibody. Furthermore, *AURKA* knockdown significantly increased LGR5 ubiquitination, as detected by an anti-ubiquitin antibody (Fig. 6C). Next, we aimed to determine which member of the ubiquitinase or deubiquitinase families might be regulated by AURKA. Through a screen of various ubiquitination-related proteins, we utilized the DUB Antibody Sampler Kit (# 8353) from Cell Signaling Technology. We identified STAMBP as a potential *AURKA*-regulated deubiquitinase, showing a strong correlation across multiple datasets (Supplementary Fig. 5A). Interestingly, *STAMBP* is significantly upregulated in gastric cancer tumors compared to normal tissues (Supplementary Fig. 5B). Moreover, *AURKA* knockdown via siRNA led to a notable decrease in STAMBP expression in AGS and STKM2 gastric cancer cell lines (Supplementary Fig. 5C, 5D). Both in vitro tamoxifen treatment and subsequent deletion of *Aurka* in organoids derived from *Krt19^CreErt^/Aurka*^flox/flox^ *Tff1*^−/−^ mice, as well as in vivo tamoxifen treatment, resulted in a significant reduction in STAMBP expression (Supplementary Fig. 6A, 6B). In addition, overexpression of *AURKA* using a Tet-On system in AGS cells significantly increased STAMBP expression (Fig. 6D), further supporting the role of AURKA in regulating STAMBP. To assess whether STAMBP regulates LGR5 ubiquitination, we knocked down *STAMBP* expression using RNA interference and assessed LGR5 ubiquitination in MG132-treated cells. LGR5 was efficiently immunoprecipitated with an anti-LGR5 antibody. Interestingly, *STAMBP* knockdown led to a marked increase in ubiquitinated LGR5 levels, as detected with an anti-ubiquitin antibody (Fig. 6E). These findings indicate that AURKA regulates LGR5 stability by modulating the activity of the STAMBP deubiquitinase. To further confirm our findings, we examined the effect of *H. pylori* infection on gastric cancer cell lines (AGS and STKM2) with or without *STAMBP* knockdown. Notably, siRNA-mediated depletion of *STAMBP* significantly reduced *H. pylori*-induced LGR5 expression, confirming that STAMBP regulates LGR5 (Fig. 6F, G). Using co-immunoprecipitation analysis following AURKA pull down in AGS cells, we found that STAMBP was effectively pulled down using the anti-AURKA antibody. Furthermore, higher levels of STAMBP coprecipitate with AURKA following *H. pylori* infection (Fig. 6H). Likewise, co-immunoprecipitation experiment in AGS cells using an anti-STAMBP antibody successfully pulled down AURKA, with higher levels of AURKA detected following *H. pylori* infection (Fig. 6I). These results indicate that AURKA and STAMBP are part of the same protein complex.

### Treatment with the AURKA inhibitor AMG-900 significantly reduced the growth of human gastric cancer tumoroids and patient-derived xenograft (PDX) tumors

De-identified gastric tumor cells were cultured as tumoroids in Matrigel and treated with the AURKA inhibitor AMG-900. After 5 days of treatment, the tumoroids exhibited a significant in size reduction compared with the untreated controls (Supplementary Fig. 7A, 7B). Consistently, both immunofluorescence (IF) and Western blot analyses revealed a marked decrease in LGR5 expression in AMG-900–treated tumoroids (Supplementary Fig. 7C–E). To confirm that AURKA could serve as a viable treatment target in gastric cancer, we subjected two patient-derived xenograft (PDX) models to treatment with AMG-900, docetaxel, or a combination of both for 28 days, starting when tumors reached 150–200 mm^3^. The combination treatment (AMG DCT) significantly reduced the tumor size in both models (*P* < 0.001, Fig. 7A, B) compared with the control group (Control). Moreover, combination therapy with AMG-900 and docetaxel significantly improved mouse survival compared to either single treatment (*P* < 0.001, Fig. 7C, D). We also investigated the molecular effects of the treatments, we performed a patient-derived xenografts (PDXs) experiment using shortened 1-week treatment regimen. In this follow-up study, immunofluorescence staining revealed that the combination therapy significantly reduced AURKA and LGR5 levels (Fig. 7E, F). Furthermore, the tumor shrinkage observed with the combination treatment was associated with decreased cell proliferation and increased apoptosis, as indicated by Ki-67 and cleaved caspase-3 staining, respectively (Fig. 7G, H). Likewise, immunofluorescence analysis of human gastric tumoroids and patient-derived xenografts (PDX) revealed a notable decrease in LGR5 and STAMBP expression following treatment with the AURKA inhibitor AMG or the combination therapy (Supplementary Fig. 8A–E). Collectively, these results strongly suggest that targeting AURKA may be a promising treatment approach for gastric cancer (Fig. 8).

## DISCUSSION

*H. pylori* infection is the leading risk factor for the development of gastric cancer. Despite extensive efforts to eradicate *H. pylori*, stomach cancer continues to pose a major global health challenge, with over 968,000 new cases and nearly 660,000 deaths reported in 2022, ranking fifth worldwide in both incidence and mortality [1]. Therefore, uncovering the molecular mechanisms that drive gastric carcinogenesis is crucial for advancing diagnostic and therapeutic strategies. In this study, we investigated the mechanistic link between *H. pylori* infection, gastric tumorigenesis, AURKA, and LGR5 regulation in gastric cancer.

Gastrointestinal cancers, particularly gastric cancer, often exhibit amplification and/or overexpression of AURKA [16, 19]. Previous studies have revealed that AURKA plays a role in activating critical oncogenic pathways, including NF-κB, β-catenin, SOX9, and STAT3, while also inhibiting the pro-apoptotic functions of p53 and p73 [15, 18, 19, 27–29]. Furthermore, we have shown that *H. pylori* infection induces AURKA expression in gastric cancer cell lines and in mice infected with the mouse-adapted *H. pylori* PMSS1 strain [15]. Although, the molecular mechanisms underlying *H. pylori*-mediated AURKA induction remain incompletely defined, we have previously shown that miR-4715-3p regulates AURKA in gastric tumorigenesis [30], suggesting that infection-driven modulation in miR-4715-3p may contribute to AURKA upregulation. Further studies are needed to elucidate the mechanisms leading to *H. pylori*-induced AURKA activation.LGR5 is a well-established marker that defines the self-renewal capacity of gastric gland stem cells [31]. Several studies have reported that LGR5-positive cell populations increase in response to *H. pylori* infection. Moreover, a tumor-resident, stem cell–like pool that drives the initiation and progression of advanced gastric cancers is enriched for LGR5 expression [8, 10, 12]. In this study, Gene Set Enrichment Analysis (GSEA) of TCGA_STAD data revealed that gastric cancer patients with high *AURKA* expression exhibited an enrichment of gene sets and pathways associated with stem cell differentiation and maintenance. Furthermore, Pearson’s correlation analysis revealed a positive association between AURKA and LGR5 expression levels, supporting a potential link between AURKA activity and stemness mediated through LGR5. Using a conditional knockout mouse model of AURKA in KRT-19-positive glands, we detected a significant reduction in both organoid growth and size. Consistent with these findings, we demonstrated that inhibition of AURKA leads to a substantial reduction in LGR5 protein levels in human gastric cancer cell lines. Conversely, overexpression of AURKA resulted in a marked increase in LGR5 protein expression.

Previous studies have established that *H. pylori* infection within the gastric antral glands induces chronic inflammation and creates a tumor-promoting microenvironment through the activation of multiple signaling pathways [32–35]. In this study, we observed that *H. pylori* infection markedly elevates LGR5 protein levels across gastric cancer cell lines, mouse gastric glands, and normal human gastric organoids. These findings agree with prior reports describing *H. pylori*–mediated induction of LGR5 expression [8, 10, 12], although the precise mechanisms remained incompletely understood. We found that AURKA knockdown is accompanied by a reduction in LGR5 expression, leading to significant impairment in gastric tumoroid formation. Moreover, pharmacological inhibition of AURKA, particularly in the presence of *H. pylori*, attenuated the LGR5-dependent increase of organoid growth derived from normal human gastric tissue. AURKA plays a critical role in driving cellular proliferation and expansion, as evidenced by AURKA silencing resulting in a reduction in LGR5 and a significant decrease in the number and size of gastric organoids. These findings were confirmed in *Aurka* CKO mice infected with *H. pylori*, showing similar inhibitory effects. Collectively, these findings suggest that AURKA plays a critical role in modulating LGR5 protein levels, allowing the expansion of the LGR5+ cell population. However, the molecular mechanisms underlying this regulation remain unclear. On this basis, we sought to investigate the direct regulatory relationship between AURKA and LGR5; however, our experiments did not provide evidence for direct transcriptional regulation of LGR5 by AURKA. These findings lead us to propose an alternative mechanism, such as protein stability, that is likely governing the relationship between AURKA and LGR5. We have demonstrated that *H. pylori* infection induces a rapid increase in endogenous AURKA protein levels, followed by upregulation of STAMBP, enhanced deubiquitinase activity, and a subsequent elevation in LGR5 expression. Notably, both genetic and pharmacological inhibition of AURKA resulted in decreased STAMBP expression, increased ubiquitination of LGR5, and a corresponding reduction in LGR5 protein levels. Additionally, we have shown a STAMBP-dependent regulation of LGR5. These findings shed new light on a previously unrecognized mechanism by which LGR5 is regulated through a protective deubiquitinating mechanism mediated by the AURKA-STAMBP axis.

The *Tff1*^−/−^ model develops neoplastic lesions in the stomach antrum that recapitulate the molecular and phenotypic characteristics of intestinal-type gastric cancer in humans [36]. We developed the *Krt19^CreErt^/Aurka*^flox/flox^/*Tff1*^−/−^ model. Using this model, we confirmed that silencing *Aurka* attenuates tumorigenesis driven by *Tff*1 knockout by reducing the proliferative capacity of gastric epithelial cells, primarily through decreased activity and expression of LGR5. Furthermore, silencing Aurka in the *Tff1*^−/−^ model led to a significant decrease in neoplastic lesions compared to control *Tff1*^−/−^ mice of matching age. These findings align with previous reports indicating that LGR5-positive cells define a population of tumor cells with self-renewal capacity in gastric cancer [12]. Furthermore, pharmacological inhibition of AURKA in patient-derived xenografts (PDXs) enhanced the anti-cancer effects of docetaxel. This therapeutic model resulted in a significant reduction in tumor burden and prolonged survival in mice. These findings are particularly significant, as current anti-cancer therapies often face major challenges due to the presence of cancer cells with stem-like properties, which possess the ability for unlimited self-renewal, multilineage differentiation, and tumor regeneration [4, 13]. LGR5 has recently emerged as a novel biomarker in multiple human malignancies, including cancers of the stomach [37], pancreas [38], liver [39], colon [40], and ovary [41]. In parallel, cancer stem cells (CSCs) are known to play a critical role in tumor initiation, maintenance, and metastasis, making them an attractive target for therapeutic intervention. Nonetheless, whether therapies directed solely against CSCs are sufficient to completely eradicate tumors remains controversial, particularly in light of the phenotypic plasticity of non-CSC populations that may enable treatment escape [42]. Based on our results, AURKA inhibition may have a substantial impact on limiting the contribution of LGR5^+^ cancer stem cells, including their roles in chemoresistance, tumor initiation, and tumor recurrence.

In summary, this study highlights the pivotal role of AURKA in gastric tumorigenesis. AURKA acts as a key signaling hub that connects *H. pylori* infection to the stabilization of the LGR5 protein by modulating its deubiquitinase, STAMBP. Targeting AURKA or STAMBP could be a promising therapeutic approach to disrupt treatment-refractory cancer cells that are enriched for stem cell–like properties in gastric cancer.

## METHODS

### Cell culture, antibodies, and reagents

The human gastric cancer cell line AGS was obtained from the American Tissue Culture Collection (ATCC) and cultured in Ham’s F-12 Nutrient Mixture (GIBCO), supplemented with 10% fetal bovine serum (FBS) (Invitrogen Life Technologies) and 1% penicillin/streptomycin (GIBCO). The human gastric cancer cell line STKM2 was generously provided by Dr. Alexander Zaika from the University of Miami and was cultured in RPMI/1640 medium supplemented with 10% FBS and 1% penicillin/streptomycin. Both cell lines were maintained in a humidified incubator at 37 °C with 5% CO_2_ and were regularly tested to ensure there was no Mycoplasma contamination. The antibodies used in this study were as follows: AURKA (D3E4Q), phospho-AURKA (Thr288) (C39D8), STAMBP (5245), Ubiquitin (E6K4Y) (Cell Signaling Technology), LGR5 (PA5-23000) (ThermoFisher), Mouse Lgr5/GPR49 Alexa Fluor^®^ 488-conjugated Antibody (RD20) (R&D), CagA (HPP-5003-9, Ango Austral Biologicals), and Actin (clone AC-15, Sigma-Aldrich). The pharmacological inhibitors, AMG-900 and MG132, were purchased from MedChem Express and Sigma Aldrich, respectively.

### Gene silencing by small interfering RNA

Cells were seeded at low density for 24 h before being transfected with Silencer^™^ Select Negative Control siRNA (ThermoFisher Scientific), Silencer^™^ Pre-Designed AURKA siRNA (ID:139201 and ID: 139203, ThermoFisher Scientific), or Silencer^™^ Pre-Designed STAMBP siRNA (ID: 17954 and ID: 18048, ThermoFisher Scientific), in 10% FBS using the Lipojet transfection kit (SignaGen Laboratories) following the manufacturer’s instructions. Whole cell lysates were prepared 48 to 72 h after transfection.

### H. pylori strains

CagA positive *H. pylori* strains (7.13, J166, and rodent-adapted PMSS1) were utilized in this study, as previously described [33]. Initially, the *H. pylori* strains were cultured on BD BBL^™^ Trypticase^™^ Soy Agar with 5% Sheep Blood plates (BD Trypticase^™^ Soy Agar II^™^, BD Biosciences) at 37 °C with 5% CO_2_ for 48 h. The strains were then passaged onto fresh Trypticase^™^ Soy Agar plates and cultured for an additional 24 h at 37 °C with 5% CO_2_. Subsequently, the *H. pylori* were cultured in Brucella broth (BD BBL^™^ Brucella Broth, BD Biosciences) supplemented with 10% fetal bovine serum (Invitrogen Life Technologies) at 37 °C with 5% CO_2_ overnight. For in vitro co-culture experiments, the 7.13 and J166 strains were co-cultured with gastric epithelial cells at a multiplicity of infection (MOI) of 50:1. For in vivo experiments, the PMSS1 strain was used to infect gastric epithelial cells in C57BL/6 mice (obtained from The Jackson Laboratory) via oral gavage, with a dosage of 1×10^9^ colony-forming units per mouse, and the infection was maintained for a duration of two weeks.

### Western blotting

In summary, cells were washed with ice-cold phosphate-buffered saline (PBS) solution. The cells were then lysed using RIPA buffer supplemented with 1× protease and phosphatase inhibitors from Santa Cruz Biotechnology. Frozen tissue samples were also lysed in RIPA buffer at −80 °C overnight. The lysates, both from cells and tissues, were sonicated and clarified through centrifugation at 16,000 xg for 10 min at 4°C. The total protein concentration of the lysates was determined using the Pierce^™^ BCA Protein Assay Kit (Pierce, Thermo Scientific). Equal amounts of protein were loaded onto a 10% SDS-PAGE gel and transferred to nitrocellulose membranes (Bio-Rad). The membranes were blocked in Tris-buffered saline Tween 20 (TBST) containing 5% bovine serum albumin (BSA) for 1 h at room temperature. Specific primary antibodies were then added to the membranes and incubated overnight at 4°C. After washing the membranes four times for 10 min each with TBST, the membranes were incubated with horseradish peroxidase-conjugated secondary antibodies (Promega) for 1 h at room temperature. Subsequently, the membranes were washed again four times for 10 min each with TBST. Finally, the blots were developed using the ECL Western blotting detection reagent (Sigma Millipore), and the signal intensity was captured using the ChemiDoc XRS+ system (Bio-Rad).

### Immunoprecipitation

The cells were washed with ice-cold PBS and then lysed in a cell lysis buffer composed of 0.5% Triton X-100, 150 mmol/L NaCl, 5 mmol/L EDTA, and 50 mmol/L Tris, supplemented with 1× protease and phosphatase inhibitors obtained from Roche. The cell lysates were clarified through centrifugation at 16000 xg for 10 min at 4°C, and the total protein concentration was determined using the Pierce^™^ BCA Protein Assay Kit (Pierce, Thermo Scientific). For immunoprecipitation, a total of 300 μg of input proteins was used per reaction. Dynabead Protein G (Invitrogen Life Sciences) was used according to the manufacturer’s instructions to couple AURKA, STAMBP, or the isotype control IgG (Santa Cruz Biotechnology Inc.) to the Dynabead Protein G. The cell lysates were then incubated with the Dynabeads-antibody complexes overnight at 4°C. Subsequently, the Dynabeads-antibody-protein complexes were pelleted using a magnet and washed six times with a washing buffer containing 0.5% Triton X-100, 150 mmol/L NaCl, 5 mmol/L EDTA, and 50 mmol/L Tris, supplemented with 1× protease and phosphatase inhibitors. The captured proteins were then eluted by incubating the samples in 1× NuPAGE^™^ LDS Sample Buffer (ThermoFisher Scientific) at 70 °C for 10 min. Finally, the samples were subjected to Western blotting using 4-20% Mini-PROTEAN^®^ TGX^™^ Precast Protein Gels (Bio-Rad).

### Quantitative real-time reverse transcriptase polymerase chain reaction (qRT-PCR)

Total RNA was extracted from cells or tissues using the RNeasy Mini Kit (Qiagen). Subsequently, cDNA was synthesized with the SuperScript cDNA Synthesis Kit (Invitrogen) according to the manufacturer’s instructions. The synthesized cDNA was diluted 10 times, and 2 μl of this dilution was used for qRT-PCR. The qRT-PCR was conducted in a 10-μl reaction volume using Bio-Rad CFX Connect Real-time System and iQ SYBR Green Supermix (Bio-Rad). Specific primers were used for the following genes: human *AURKA*: forward, 5′-GCTGGAGAGCTTAAAATTGCA-3′ and reverse, 5′-TTTGTAGGTCTCTTGGTATGTG-3′; human *LGR5*: forward, 5′-CACCTCCTACCTAGACCTCAGT-3′ and reverse, 5′-CGCAAGACGTAACTCCTCCAG-3′; human *HPRT1*: forward, 5′-ACCCTTTCCAAATCCTCAGC-3′ and reverse, 5′-GTTATGGCGACCCGCAG-3′; mouse *Aurka*: forward, 5′- CTGGATGCTGCAAACGGATAG-3′ and reverse, 5′- CGAAGGGAACAGTGGTCTTAACA-3′; mouse *Lgr5*: forward, 5′-CAGCCTCAAAGTGCTTATGCTG-3′ reverse, 5′-TGGCACGTAACTGATGTGG -3; mouse *Hprt*: forward, 5′- CCTAAGATGAGCGCAAGTTGAA-3′ and reverse, 5′- CCACAGGACTAGAACACCTGCTAA-3′. The threshold cycle number was determined using Bio-Rad CFX Manager^™^ software version 3.0. Reactions were conducted in triplicate, and the threshold cycle numbers were averaged. The data were normalized to the HPRT1 housekeeping gene.

### Gene set enrichment analysis (GSEA)

GSEA was utilized to analyze the AURKA-associated signaling pathways. The “limma” package was used to identify the differentially expressed genes (DEGs) between AURKA high-expression samples compared to AURKA low-expression samples in TCGA and GEO datasets (GSE122401, GSE54129, GSE66229, GSE15459, GSE110875, GSE26899, GSE26901). The Normalization Enrichment Score (NES) and false discovery rate (FDR) of each gene set were calculated using the “clusterProfiler” package (v3.12.0) [43]. A gene set was considered significant when the FDR was less than 25%. Reference gene sets, including hallmark gene sets, and GO biological processes gene sets, were downloaded from MSigDB (https://www.gsea-msigdb.org/). All data were analyzed using R software (R 3.6.1; https://www.r-project.org/). To evaluate the overall association between AURKA, LGR5, and STAMBP expression across multiple independent datasets, a meta-analysis of correlation coefficients was conducted using the “metafor” package in R. Each dataset provided an estimate of the Pearson correlation coefficient (r) between the two genes of interest, along with the corresponding sample size (n). Before meta-analysis, the correlation coefficients were transformed using Fisher’s r-to-z transformation to stabilize variance and normalize the distribution: $z=\frac{1}{2}\text{ln}(\frac{1+r}{1-r})$, The standard error (SE) for each transformed correlation was calculated as ${\mathrm{SE}}_{z}=\frac{1}{\sqrt{n-3}}$. A random-effects model was fitted using the rma() function in the metafor package, incorporating between-study heterogeneity using the DerSimonian-Laird method. The model estimated the overall effect size (Fisher’s z) and 95% confidence interval, which were subsequently back transformed to correlation coefficients for interpretation. Statistical heterogeneity across studies was assessed using Cochran’s Q statistic and the I^2^ statistic. An I^2^ value > 50% was considered to indicate substantial heterogeneity.

### Immunofluorescence

For paraffin-embedded tissue sections, the slides were deparaffinized, followed by antigen retrieval in a pressure cooker for 30 min in 1X-TE buffer (pH 9). Blocking was performed for 1 h at room temperature in 5% BSA (bovine serum albumin) in 1X PBS. Next, the tissue sections were incubated with a combination of three primary antibodies: anti-LGR5 (1:100), anti-STAMBP (1–100), and anti-AURKA (1:100) overnight at 4 °C. The following day, sections were washed and incubated with secondary antibodies, donkey anti-mouse IgG conjugated to Alexa Fluor 488 and donkey anti-rabbit IgG conjugated to Alexa Fluor 568 (Thermofisher), diluted in blocking buffer (1:500) for 1 h at room temperature. For nuclear staining, sections were washed three times and incubated with Hoechst (10 μg/ml) for 10 min. The tissue sections were mounted, and images were captured using the Leica Stellaris 5 confocal microscope.

For immunofluorescence on cells, cells were seeded for 24 h prior to transfection with Silencer^™^ Select Negative Control siRNA (ThermoFisher Scientific) or Silencer^™^ Pre-Designed AURKA siRNA for a total of 72 h. AURKA or control siRNA were plated in the 8-well chambers and co-cultured with *Helicobacter pylori* strains 7.13 and J166 for 6 h at a multiplicity of infection (MOI) of 50:1 when applicable. Cells were washed with PBS and fixed with 4% paraformaldehyde for 20 min at room temperature. After a permeabilization step, cells were blocked using 10% nonimmune goat serum blocking solution (Invitrogen) for 1 h at room temperature and incubated with primary antibodies anti-LGR5 (1:100), anti-STAMBP (1-100) or anti-AURKA (1:100) overnight at 4 °C. After 4× washes with washing buffer (PBS-3% Bovine Serum Albumin-0.1% Triton X100), cells were incubated with donkey anti-rabbit/anti-mouse IgG secondary antibodies conjugated to AF488 and AF568 (Invitrogen) and incubated for 1 h at room temperature. Cells were then washed 4× times with washing buffer and mounted with Vectashield Mounting Medium containing DAPI (4′,6-diamidino-2-phenylindole) (Vector Laboratories). Images were acquired using a Leica Stellaris 5 Confocal Microscope and analyzed using the Leica Application Suite X (LAS X).

### Animal care

All animal studies followed our approved protocol (UM-23-110) by the Institutional Animal Care and Use Committee of the University of Miami (Miami, FL).

### Mice infection with *H. pylori*

Mice were infected with *H. pylori* following the guidelines outlined in the Guide for the Care and Use of Laboratory Animals published by the National Institutes of Health. All procedures were approved by the Institutional Animal Care and Use Committee (IACUC) at the University of Miami. C57BL/6 mice obtained from Charles River Laboratories were divided into two groups (8 per group) for either *H. pylori* infection or control (PBS). The study was designed to provide adequate sensitivity (*n* = 32; *n* = 8/group in a 2×2 factorial design), under standard ANOVA assumptions to detect large, biologically relevant effects consistent with prior studies of AURKA-deficient gastric epithelium challenged by *H. pylori*. The design provided 80% power (α = 0.05) to detect main-effect differences of approximately Cohen’s d ≈ 1.02 ( ≈ 1 SD shift) and an interaction effect of Cohen’s f ≈ 0.53, a magnitude aligned with known genetic × infection divergences in epithelial injury, cytokine induction, and proliferative responses. Additionally, *Krt19*^CreErt^/*Aurka*^flox/flox^ mice [15] aged 8–10 weeks were divided into four groups (8-9 per group) for treated with or without Tamoxifen (50 mg/kg, IP) for 10 days. In the case of *H. pylori* infection, mice received orogavage of either PBS (uninfected control) or the mouse-adapted wildtype *H. pylori* strain PMSS1 (109 CFU/mouse) at the age of 10–12 weeks [44]. Subsequently, the mice were euthanized two weeks post challenge, and gastric tissues were collected for pathology, Western blots, and real-time PCR analyses. All histological changes were evaluated by our gastrointestinal pathologist (OGM). All mice used in the experiment were included in the data analysis; no inclusion or exclusion criteria were applied. Mice were randomly allocated to the two experimental groups using simple randomization, ensuring equal probability of assignment for each animal given their matched age and background. No formal stratified randomization was performed, as baseline covariates were already similar across the cohort.

### Mouse generation and experimental design

Compound mutant mice were generated by crossing *Krt19*^CreErt^/*Aurka*^−ox/−ox^ mice [15] with *Tff1^−/−^* mice [45] to produce *Krt19*^CreErt^/*Aurka*^flox/flox^*Tff1^−/−^* mice. This model allows for tamoxifen-inducible, stomach epithelium-specific AURKA ablation through Cre-mediated recombination in KRT19-expressing cells. Age-matched *Krt19*^CreErt^/*Aurka*^flox/flox^*Tff1^−/−^* mice (12–14 weeks old) were assigned into two experimental groups (*n* = 8–10/group). (Control *n* = 9; Tamoxifen *n* = 8), the two-group comparison yields 80% power to detect a large effect of Cohen’s d ≈ 1.46, whereas more modest effects (d ≈ 0.6–0.8) would be underpowered and should be interpreted cautiously (e.g., ~34% power at d = 0.8 and ~49% at d = 1.0). This framing reflects ethical and logistical constraints on additional animals and appropriately aligns the analytic expectations with the likelihood of robust phenotypic changes in AURKA CKO models within the Tff1-deficient background. The tamoxifen-treated group received 50 mg/kg tamoxifen (intraperitoneal injection) daily for 10 consecutive days. Controls received the vehicle alone. Three months post-treatment, mice were euthanized following the IACUC-approved protocol (UM 23-110). Gastric tissues were collected and processed for histopathological analysis (formalin-fixed, paraffin-embedded), molecular studies (snap-frozen in liquid N_2_, stored at −80°C), protein analysis (western blotting), and gene expression profiling (quantitative RT-PCR). All mice used in the experiment were included in the data analysis; no inclusion or exclusion criteria were applied. All mice used in the experiment were included in the data analysis; no inclusion or exclusion criteria were applied. Mice were randomly allocated experimental groups using simple randomization, ensuring equal probability of assignment for each animal given their matched age and genetic background. No formal stratified randomization was performed, as baseline covariates were already similar across the cohort.

### Patient-derived xenografts (PDX)

De-identified PDX models of human gastric carcinoma were established using a previously validated protocol [46]. Uniform tumor fragments were subcutaneously implanted into both flanks of immunodeficient mice, and tumor growth was closely tracked over time. Once the mean tumor volume reached approximately 150 mm^3^, mice were randomly divided into four groups (*n* = 6 per group): vehicle control, docetaxel alone, AMG-900 alone, and combined AMG-900 and docetaxel treatment. Docetaxel was dissolved in phosphate-buffered saline (PBS) and administered intraperitoneally at 10 mg/kg once weekly for a total of four weeks. AMG-900 was prepared in a solution of 2% dimethyl sulfoxide (DMSO), 2% Tween-80, and 96% sterile water, and delivered intraperitoneally at 15 mg/kg three times per week for four weeks. Tumor length and width were recorded three times per week with a digital caliper, and tumor volume was determined using the formula: volume (mm^3^) = ½ × (width)^2^ × length. Mouse body weights were measured weekly to assess potential systemic toxicity. Following treatment, mice were monitored for survival until tumors reached a volume of 1000 mm^3^, at which point mice were euthanized according to humane endpoints. Survival data were analyzed using Kaplan–Meier estimation, with statistical differences assessed by the log-rank (Mantel–Cox) test.

### Primary gastric epithelial cells and 3D organoid cultures

Short-term cultures of primary gastric epithelial cells were prepared following our standard protocol [47]. 3D organoid tissue cultures were prepared based on the protocol by Barker et al. [31]. Mouse stomachs were surgically excised, opened, and rinsed with HBSS. The gastric antrum was sectioned and incubated in 5 ml of 5 mM EDTA for 2 h at 4 °C with gentle agitation. After incubation, EDTA was replaced with 5 ml of chelation buffer, and the tissue was shaken vigorously for approximately 2 min to release gastric glands. The resulting suspension was passed through a 70 μm nylon mesh cell strainer (Becton Dickson, Franklin Lakes, NJ) to remove muscle tissue, followed by centrifugation at 150 × g for 5 min. The gland pellet was embedded in an extracellular matrix hydrogel (Trevigen, Inc., Gaithersburg, MD) enriched with IntestiCult Organoid Growth Medium (StemCell Technologies, Vancouver). By day 3, glands formed mature organoids, which were subcultured every 12 days. A 0.1 μM tamoxifen solution was introduced into the culture medium for 48 h, after which the medium was replaced with fresh medium. Organoids were recovered from Matrigel using the Cell Recovery Solution reagent (Gibco) and processed into standard formalin-fixed, paraffin-embedded tissue specimens.

### Establishment of patient-derived gastric organoids

De-identified normal and gastric cancer tissue samples were obtained through the Biospecimen Shared Resource (BSSR) at the University of Miami (Miami, FL), with study approval granted by the Institutional Research Ethics Committee. Informed consent was secured from all participants by the BSSR prior to inclusion. The research adhered to the ethical standards set forth in the Declaration of Helsinki. Freshly excised tissue samples (3–5 mm) were placed in human organoid growth medium (IntestiCult Organoid Growth Medium, Stemcell Technologies, RRID: SCR_013642; 06010) and maintained at 4 °C until processing, which was performed within 1 h of surgical removal to optimize organoid formation. Tissue samples were processed as previously described [22]. Specifically, tumor fragments were sectioned into 0.1–0.5 mm pieces using sterile scalpels, washed with medium to remove debris, and primarily used for organoid culture. Remaining fragments were either snap-frozen in liquid nitrogen for RNA or protein extraction or fixed in 10% formalin for histologic evaluation. Organoid culture preparation were established according to the protocol described by Mahe et al. [48]. Briefly, minced tumor tissue was incubated in 2 mL of 5 mg/mL collagenase type II (Gibco; Thermo Fisher Scientific, RRID: SCR_008452, catalog no. 17101-015) dissolved in Advanced DMEM/F12 (Gibco; Thermo Fisher Scientific, catalog no. 12634-010) for 45 min at 37 °C. The digest tissue was then treated with TrypLE (Thermo Fisher Scientific, catalog no. 12604021) before being resuspended in DMEM/F12. To eliminate debris and achieve single-cell suspensions, the resuspension was strained through a 40 μm cell strainer. Approximately 5,000 passage-zero cells were cryopreserved in FBS (Thermo Fisher Scientific, catalog no. A3160402) supplemented with 10% DMSO (Sigma-Aldrich, RRID: SCR_008988; catalog no. D5879) and stored in liquid nitrogen as a patient-derived cell stock. Cell viability was assessed using trypan blue staining, and viable cells were suspended in growth factor–reduced Matrigel (Corning Life Sciences; catalog no. 354230) at a concentration of 20,000 cells/50 μL of 90% Matrigel. Each 50 μL droplet was plated into the center of a 24-well plate and allowed to polymerize at 37 °C for 30 min prior to adding prewarmed (37 °C) organoid growth medium (IntestiCult Organoid Growth Medium, Stemcell Technologies, RRID: SCR_013642; #06010). Organoids were harvested and passaged every 10-14 days depending on growth characteristics. For collection, organoids were gently recovered with ice-cold Cell Recovery Solution, enzymatically dissociated with TrypLE, and reseeded at split ratios of 1:2 or 1:3 in 90% Matrigel to continue culture.

### Treatment of patient-derived gastric organoids

As previously described [36], human organoid growth medium (IntestiCult^™^ Organoid Growth Medium, Stemcell Technologies; RRID:SCR_013642; Cat. #06010) was supplemented into cell suspensions obtained from enzymatically digested human tissue samples, in the presence or absence of test compounds, and each condition was plated in triplicate. The organoids were cultured at 37°C in a humidified incubator with 5% CO_2_, and the culture medium was replenished every 2–3 days. The organoids were transduced with the recombinant adenoviral vector pAV[Exp]-EGFP-CMV>h*AURKA* (VB900148-9083acy) at a multiplicity of infection (MOI) of 25 overnight. The culture medium was then replaced after 24 h. AMG-900 was dissolved in dimethyl sulfoxide (DMSO) to achieve a final concentration of 0.1% (v/v). To assess the effects of the drug treatment on organoid proliferation and viability, the diameter and total count were quantified after 4–5 days of treatment. A minimum of 50 organoids per condition were measured to determine the mean diameter using the Perfect Screen Ruler software. Organoids were counted manually using bright-field microscopy under a 20× objective.

### Statistical analysis

Comparison of survival (percent) curves was done using Log-rank (Mantel-Cox) test.

Data are expressed as the mean ± SE or ± SEM for each condition. Statistical significance was determined using the GraphPad Prism statistical software, and the nonparametric Mann-Whitney test was used for comparisons not assuming normal distribution. Differences with *p* values ≤ 0.05 are considered significant.

## Supplementary Material

Supplementary Figures

Supplementary Tables

The online version contains supplementary material available at https://doi.org/10.1038/s41418-026-01810-w.

## Figures and Tables

**Fig. 1:**
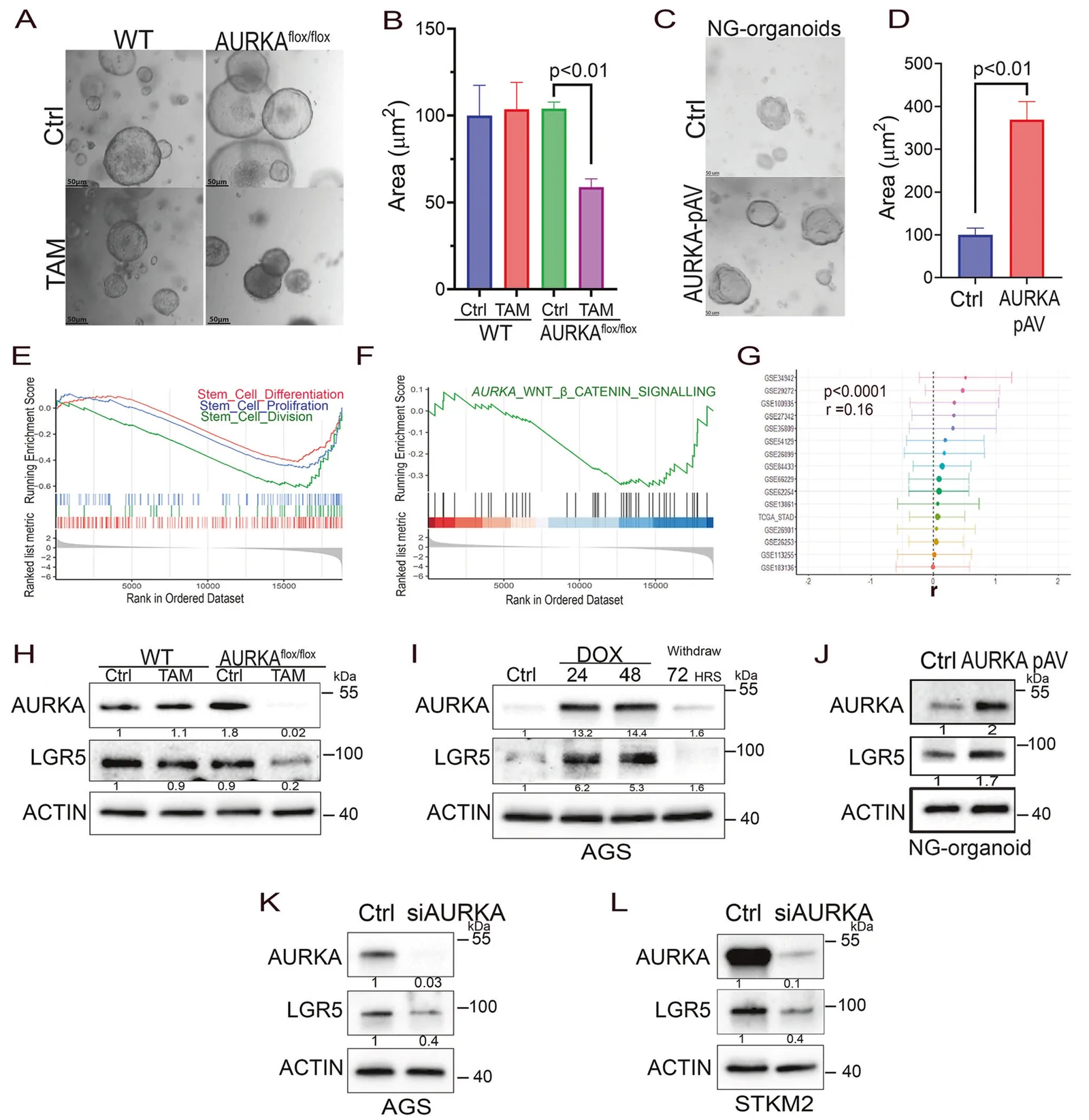
AURKA regulates organoid growth by inhibiting LGR5. **A**, **B** Gastric organoids were established from *Krt19^CreErt^/Aurka*^flox/flox^ mice and treated with tamoxifen (TAM) or left untreated (Ctrl) in vitro. Representative images are shown (scale bars, 50 μm) and the size is quantified as the mean ± SEM of area (μm^2^) (*n* = 15). **C**, **D** Gastric organoids were established from a normal human stomach transduced with pAV[Exp]-EGFP-CMV>h*AURKA*. Representative images are shown (scale bars, 50 μm) and organoid size was quantified as the mean ± SEM of the area (μm^2^) (*n* = 6). **E**, **F** Gene set enrichment analysis (GSEA) was preformed using human samples with high versus low *AURKA* expression from TCGA and GEO datasets, showing enrichment of stem cell and *β-CATENIN* signatures. **G** Pearson’s correlation test comparing *AURKA* and *LGR5* expression in human gastric cancer GEO datasets. **H** Western blot analysis of AURKA and LGR5 protein levels in gastric organoids established from *Krt19^CreErt^/Aurka*^flox/flox^ mice that received tamoxifen (TAM) or left untreated (Ctrl) in vitro. **I** Western blot analysis of AURKA and LGR5 expression in AGS cells following AURKA overexpression. **J** Western blot analysis of AURKA and LGR5 expression in human gastric organoids transduced with pAV[Exp]-EGFP-CMV>hAURKA. **K**, **L** Western blot analysis of AURKA and LGR5 protein levels in AGS and STKM2 cells, both with and without AURKA knockdown. All data shown are representative of 3 independent experiments.

**Fig. 2:**
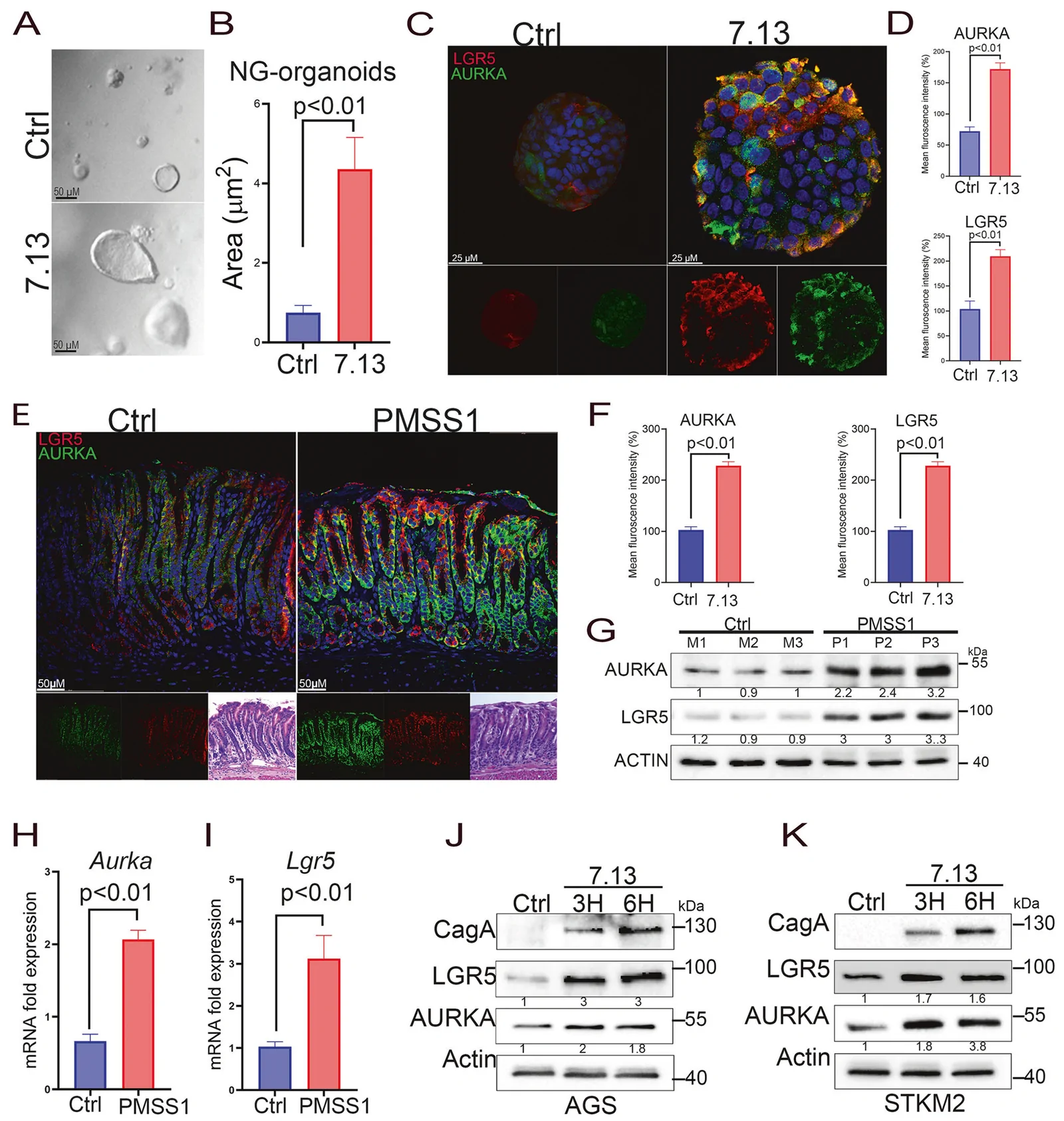
*H. pylori* infection induces AURKA and LGR5 expression. **A**, **B** Gastric organoids established from normal human stomach tissue and infected with *H. pylori* strain 7.13. Representative images are shown (scale bars, 50 μm) and organoid size was quantified as the mean ± SEM area (μm^2^) (*n* = 20). **C**, **D** Immunofluorescence staining of AURKA (green) and LGR5 (red) in human gastric organoids infected with *H. pylori* strain 7.13. Representative images are shown (scale bars, 50 μm) (*n* = 4). **E**, **F** Immunofluorescence staining of AURKA (green) and LGR5 (red) in mice infected or not with *H. pylori* strain PMSS1. Representative images are shown, along with H&E staining demontrating histological features of murine gastric mucosa (scale bars, 50 μm) (*n* = 5). **G** Western blot analysis of AURKA and LGR5 in the antrum of mice infected or not with PMSS1. **H**, **I** qRT-PCR analysis of *Aurka* and *Lgr5* mRNA in mice infected with *H. pylori* strain PMSS1 (*n* = 5). **J**, **K** Western blot analysis of CagA, AURKA, and LGR5 in AGS and STKM2 cells following *H. pylori* infection (7.13). All data shown are representative of 3 independent experiments.

**Fig. 3:**
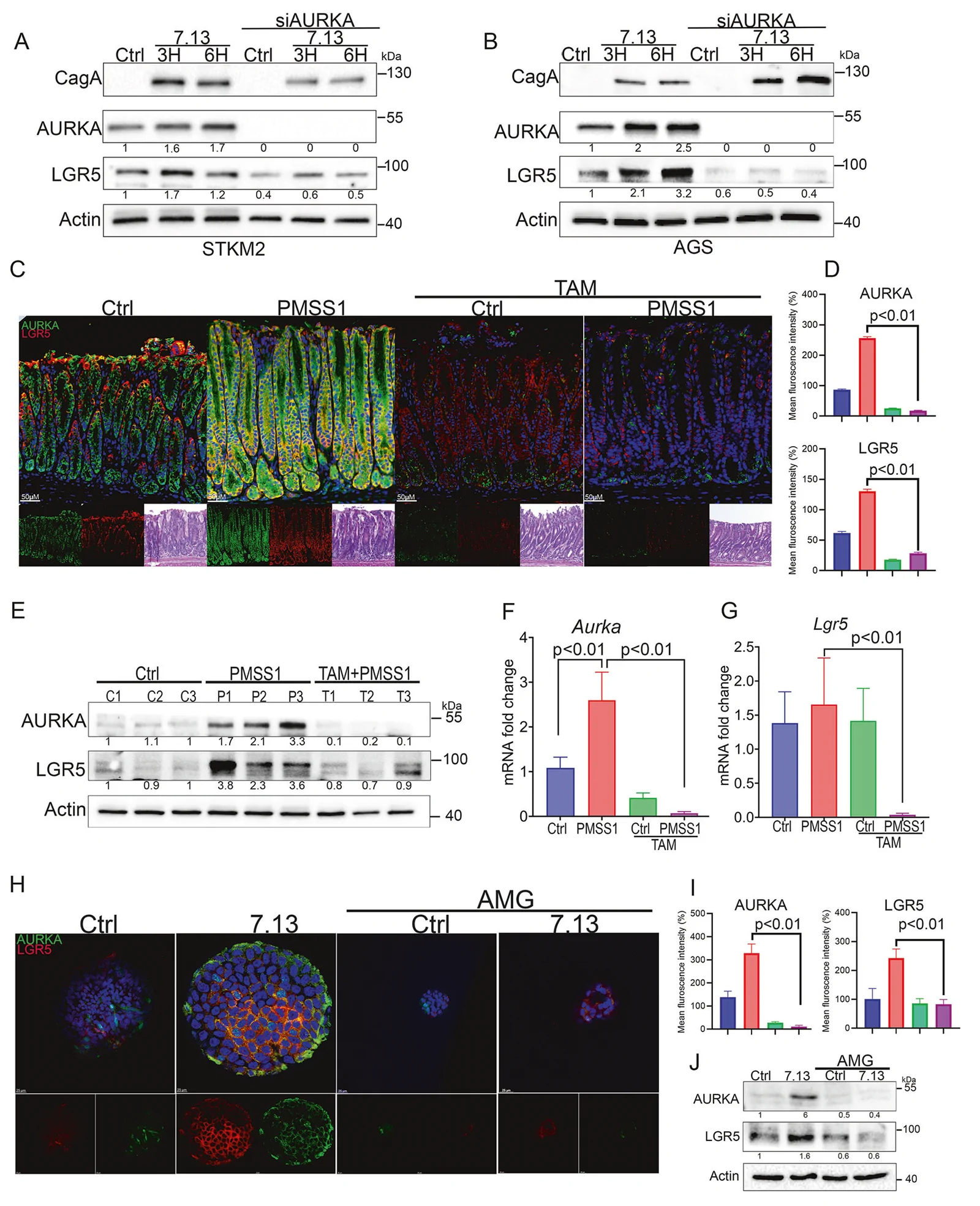
AURKA knockdown suppresses *H. pylori*-dependent LGR5 induction. **A, B** Western blot analysis of CagA, AURKA, and LGR5 with or without AURKA knockdown following *H. pylori* infection in AGS and STKM2 cells. **C**, **D** Representative images of immunofluorescence staining for AURKA (green) and LGR5 (red) expression in *Krt19^CreErt^/ Aurka*^flox/flox^ mice treated with tamoxifen (TAM) or left untreated (Ctrl) followed by *H. pylori* infection (PMSS1) (*n* = 4). Representative H&E stained showing the histological features of gastric mucosa are also shown (scale bars, 50 μm) (*n* =4). **E** Western blot analysis of AURKA and LGR5 in *Krt19^CreErt^/ Aurka*^flox/flox^ mice treated with tamoxifen (TAM) or left untreated (Ctrl) followed by *H. pylori* infection (PMSS1). **F**, **G** qRT-PCR analysis of *Aurka* and *Lgr5* mRNA in *Krt19^CreErt^/ Aurka*^flox/flox^ mice that received tamoxifen (TAM) or not (Ctrl) followed by *H. pylori* infection (PMSS1) (*n* = 8). **H**, **I** Representative images of immunofluorescence staining for AURKA (green) and LGR5 (red) in human gastric organoids infected with *H. pylori* (7.13) with or without AMG-900 treatment (scale bars, 25 μm) (*n* = 8). **J** Western blot analysis of AURKA and LGR5 in human gastric organoids infected with *H. pylori* (7.13) with or without AMG-900 treatment. All data are representative of 3 independent experiments.

**Fig. 4:**
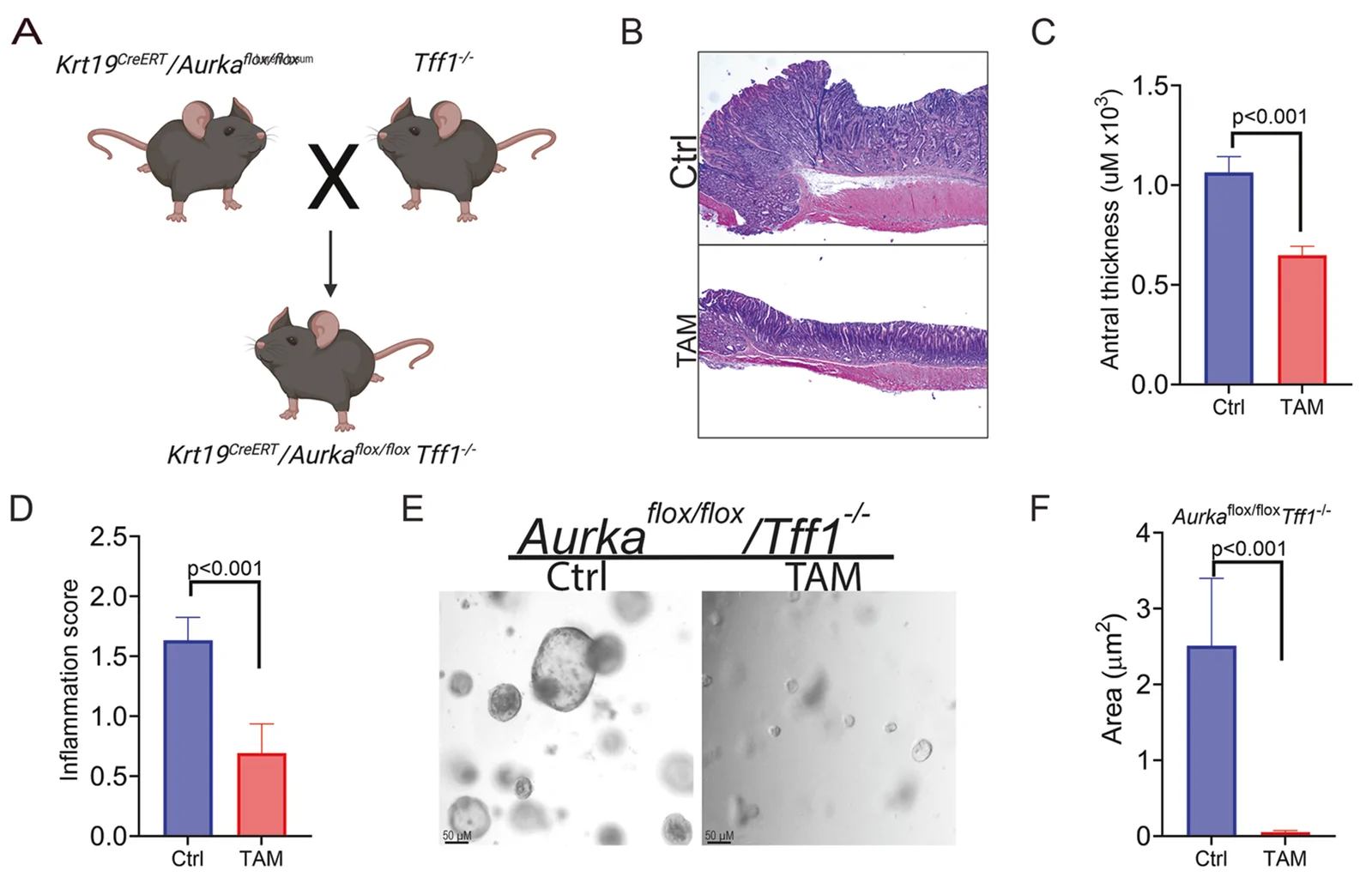
AURKA knockdown suppresses tumorigenesis in the *Tff1−/−* mouse model of gastric cancer. **A** Illustration of the *Krt19^Cre^/ Aurka*^flox/flox^*Tff1*^−/−^ knockout mouse model. **B** Representative H&E images of the gastric gland of *Krt19*^Cre^/*Aurka*^flox/flox^*Tff1*^−/−^ mice that received tamoxifen (TAM) or not (No TAM) (*n* = 8). **C**, **D** Quantification of the thickness and inflammation score in gastric glands of *Krt19*^Cre^/*Aurka*^flox/flox^*Tff1*^−/−^ mice that received tamoxifen (TAM) or not (No TAM controls) (*n* = 8). **E**, **F** Gastric organoids derived from *Krt19*^Cre^/*Aurka*^flox/flox^*Tff1*^−/−^ mice that received tamoxifen (TAM) or were untreated (Ctrl). Representative images are shown (scale bars, 50 μm) and organoid size was quantified as the mean ± SEM area (μm^2^) (*n* = 8).

**Fig. 5:**
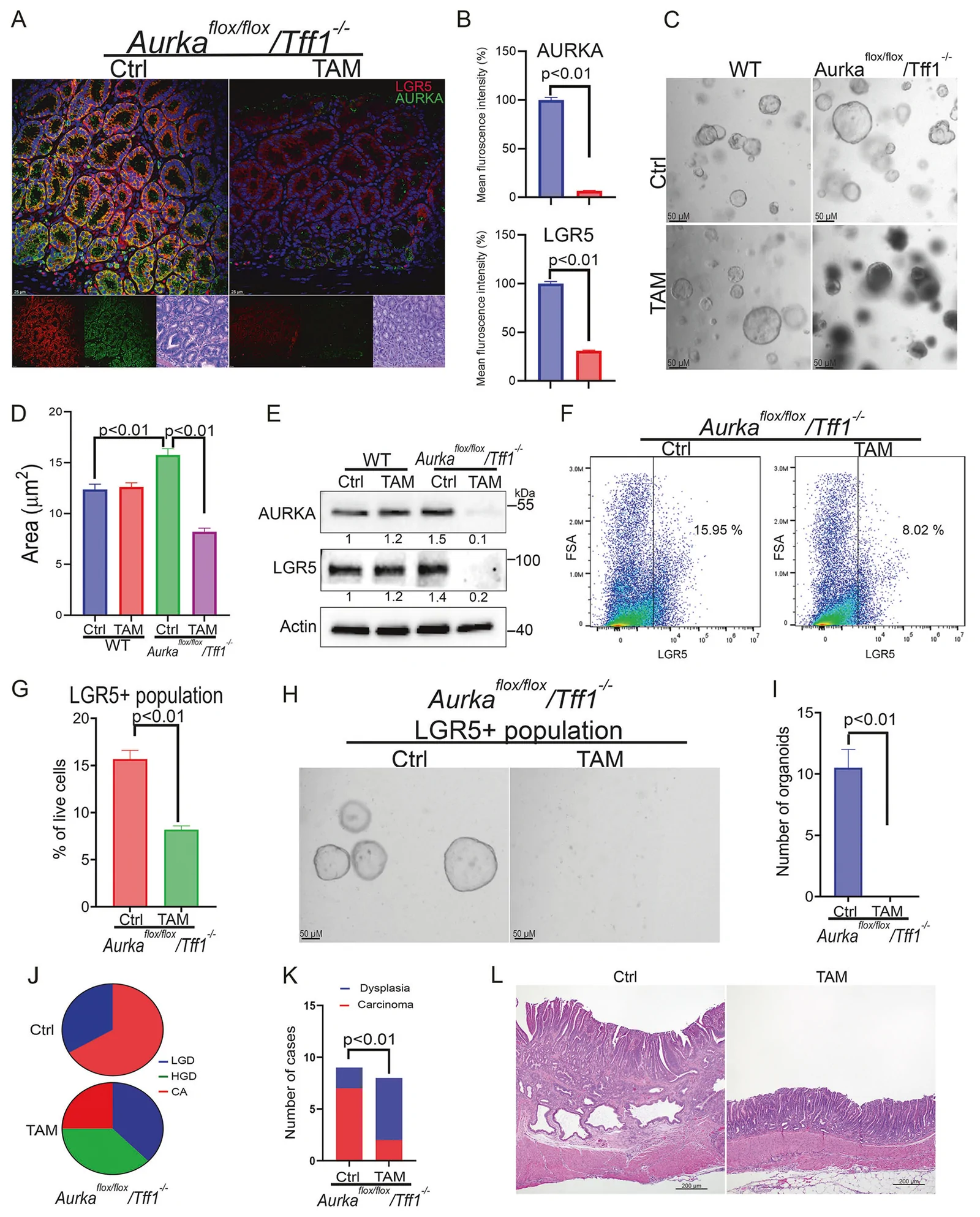
Genetic deletion of *Aurka* reduces the population of LGR5+ cells in gastric tumors. **A**, **B** Representative images of immunofluorescence staining for AURKA (green) and LGR5 (red) in *Krt19^CreErt^/Aurka*^flox/flox^*Tff1*^−/−^ mice that received tamoxifen (TAM) or not (Ctrl) (*n* = 5). H&E stainings of representative histological features of gastric mucosa are shown (scale bars, 25 μm) (*n* = 5). **C**, **D** Gastric organoids established from WT and *Krt19^CreErt^* /*Aurka*^flox/flox^*Tff1*^−/−^ mice that received tamoxifen (TAM) or not (No TACtrlM) in vitro. Representative images are shown (scale bars, 50 μm) and size quantified as the mean ± SEM of the area (μm^2^) (*n* = 6). **E** Western blot analysis of AURKA and LGR5 in gastric organoids established from WT and *Krt19^CreErt^*/ *Aurka*^flox/flox^*Tff1*^−/−^ mice that received tamoxifen (TAM) or not (Ctrl) in vitro. **F**, **G** Representative FACS analysis of LGR5 positive cells proportion in WT and *Krt19^CreErt^*/*Aurka*^flox/flox^*Tff1*^−/−^ mice that received tamoxifen (TAM) or not (Ctrl) (*n* = 3). **H**, **I** Gastric organoids established from LGR5 positive population sorted from the stomach of WT and *Krt19^CreErt^ Aurka*^flox/flox^*Tff1*^−/−^ mice that received tamoxifen (TAM) or not (Ctrl). Representative images are shown (scale bars, 50 μm) and size quantified as the mean ± SEM of the area (μm^2^) (*n* = 8). **J**, **K** Quantification of dysplasia and hyperplasia scores in the gastric glands of *Krt19^Cre^/ Aurka*^flox/flox^*Tff1*^−/−^ mice that received tamoxifen (TAM) or not (Ctrl) (*n* = 8). **L** Representative H&E images of the gastric gland of *Krt19^Cre^*/ *Aurka*^flox/flox^*Tff1*^−/−^ mice that received tamoxifen (TAM) or not (No TAM) (*n* = 8).

**Fig. 6:**
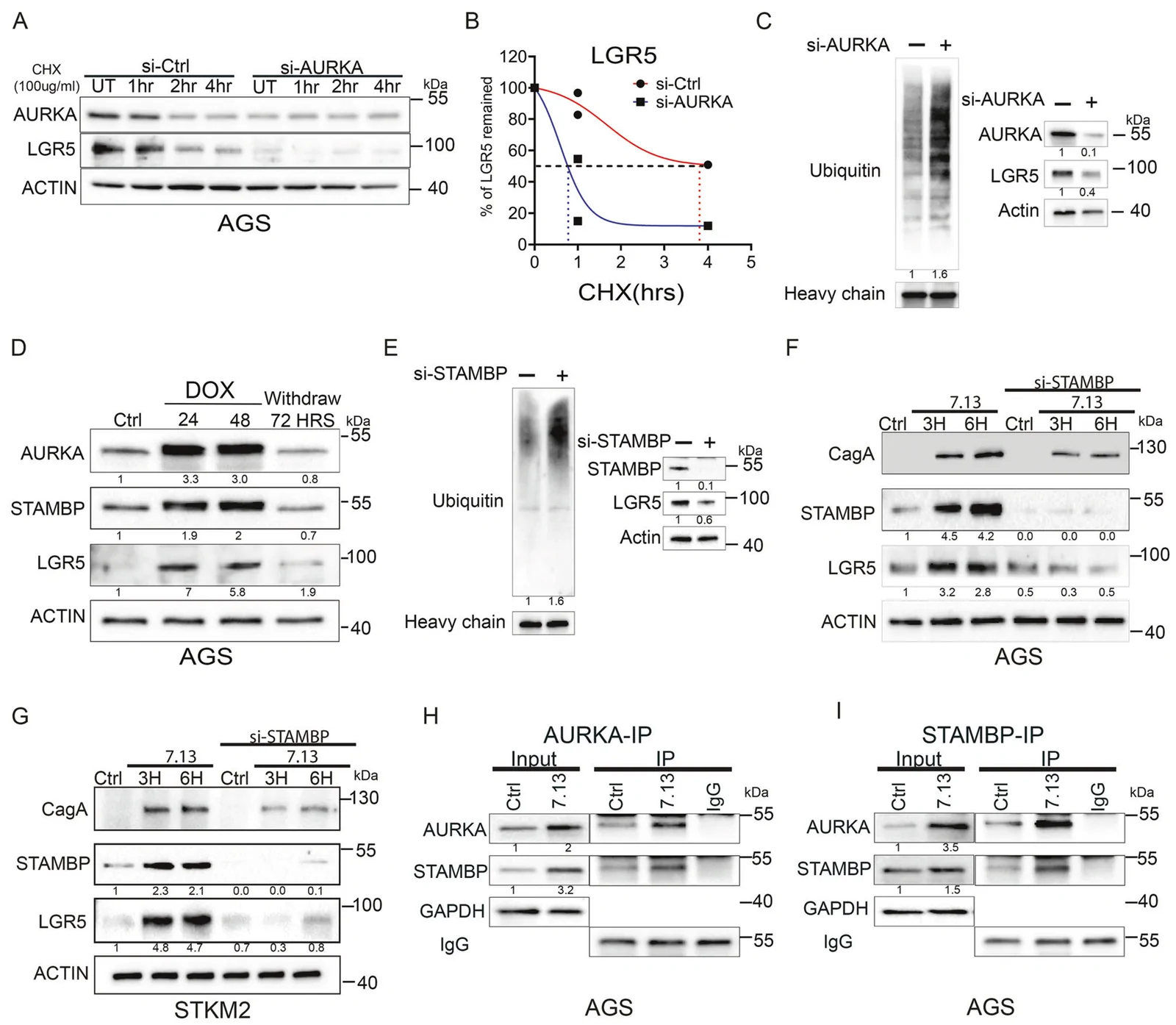
AURKA regulates the stability of LGR5 by modulating its ubiquitination. **A**, **B** CHX assay in AGS cells with or without AURKA knockdown: (**A**) Western blot analysis of LGR5 stability over the indicated time points. **B** Quantification of LGR5 protein stability in the presence or absence of AURKA knockdown. **C** Ubiquitination assay using an anti-Ubiquitin antibody following the immunoprecipitation of LGR5 with/without AURKA knockdown. ACTIN and the heavy chain (HC) were used as loading controls. **D** Western blot analysis of AURKA, STAMBP, and LGR5 expression in AGS cells following AURKA overexpression. **E** Ubiquitination assay using an anti-Ubiquitin antibody following the immunoprecipitation of LGR5 with/without STAMBP knockdown. ACTIN and the heavy chain (HC) were used as loading controls. **F**, **G** Western blot analysis of CagA, AURKA, and LGR5 with or without STAMBP knockdown followed by *H. pylori* infection in AGS and STKM2 cells. **H**, **I** Immunoprecipitation assay using anti-AURKA or anti-STAMBP antibodies. Anti-Immunoglobulin (IgG) was used as a negative control, and anti-GAPDH was used as a loading control. All data shown are representative of 3 independent experiments.

**Fig. 7:**
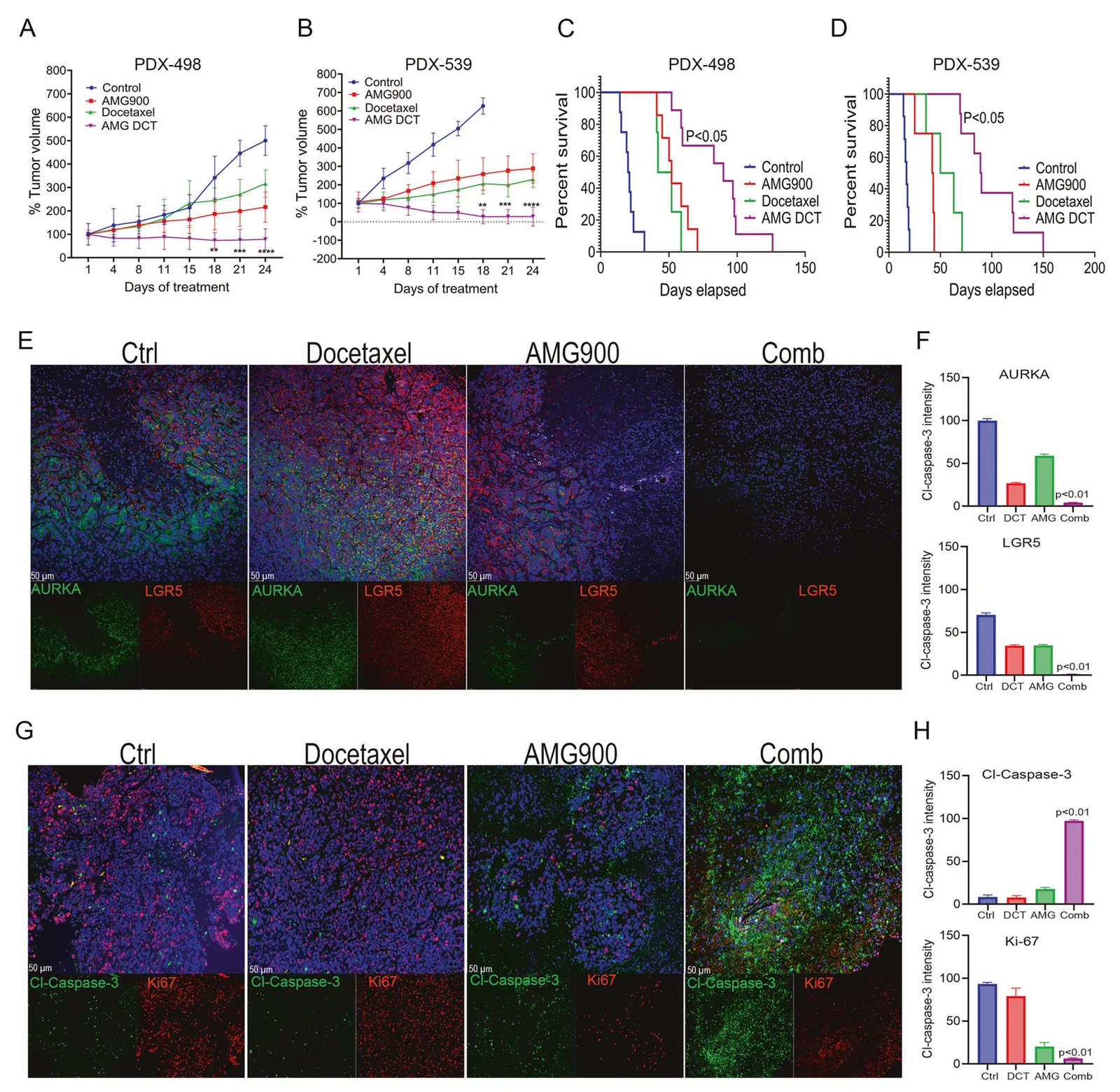
Treatment with the AURKA inhibitor AMG-900 significantly reduced the growth of human gastric cancer tumoroids and patient-derived xenograft (PDX) tumors. **A**, **B** Average tumor volume of PDXs treated with or without Docetaxel (10 mg/kg), AMG-900 (15 mg/kg), or the combination treatment for 28 days (*n* = 6 mice/group; data is presented as mean ± SD; *p* < 0.05) (*n* = 5). **C**, **D** Kaplan–Meier survival curves for the same PDXs following the treatment endpoint (*n* = 5). **E**, **F** Representative immunofluorescence staining images of AURKA (green) and LGR5 (red) in the PDX tumor tissues. DAPI was used for nuclear staining (*n* = 3). **G**, **H** Representative immunofluorescence staining images of the cell proliferation marker Ki67 (green) and the apoptosis marker cleaved caspase 3 (red) in the PDX tumor tissues. DAPI was used for nuclear staining (*n* = 3).

**Fig. 8:**
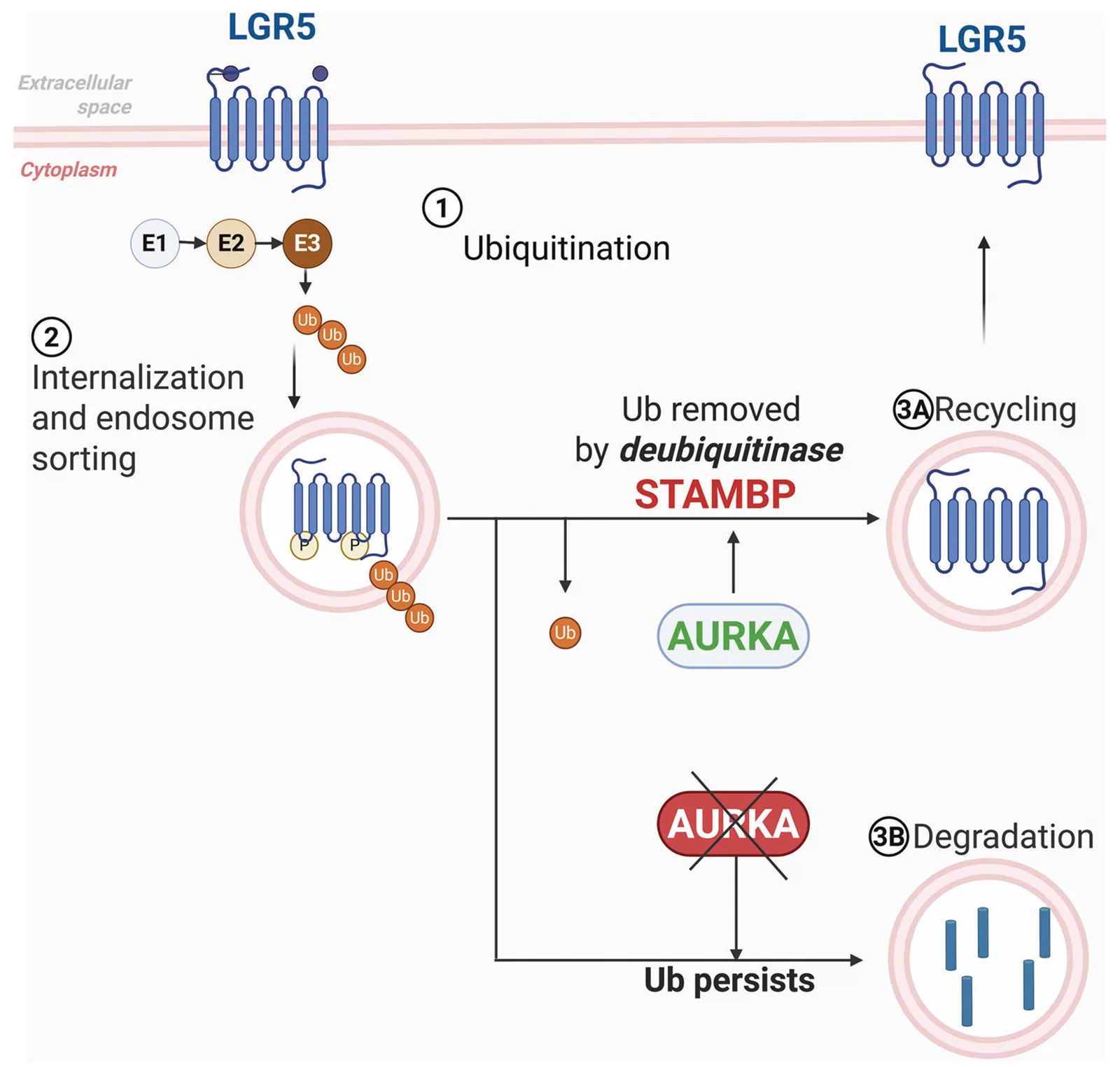
Proposed model of the AURKA–STAMBP–LGR5 axis in gastric tumorigenesis. *H. pylori* infection induces *AURKA* expression, which promotes STAMBP-mediated deubiquitination and stabilization of LGR5, leading to expansion of LGR5-positive stem-like cells, enhanced organoid growth, and gastric tumorigenesis. Genetic deletion or pharmacologic inhibition of AURKA increases LGR5 ubiquitination and degradation, suppressing tumor growth and enhancing the therapeutic efficacy of docetaxel.

## Data Availability

All data generated or analyzed during this study are included in this published article and its supplementary information.
