# Supplementary material for: AURKA regulates LGR5 in response to Helicobacter Pylori infection by modulating its deubiquitination
Source: Cell Death Differ. Author manuscript; Available in PMC 2026 Oct 1. (PMC13628944; doi:10.1038/s41418-026-01810-w)
Supplement: Supplementary file 1 [file NIHMS2210525-supplement-Supplementary_Figures.pdf]

Supplementary figure 1

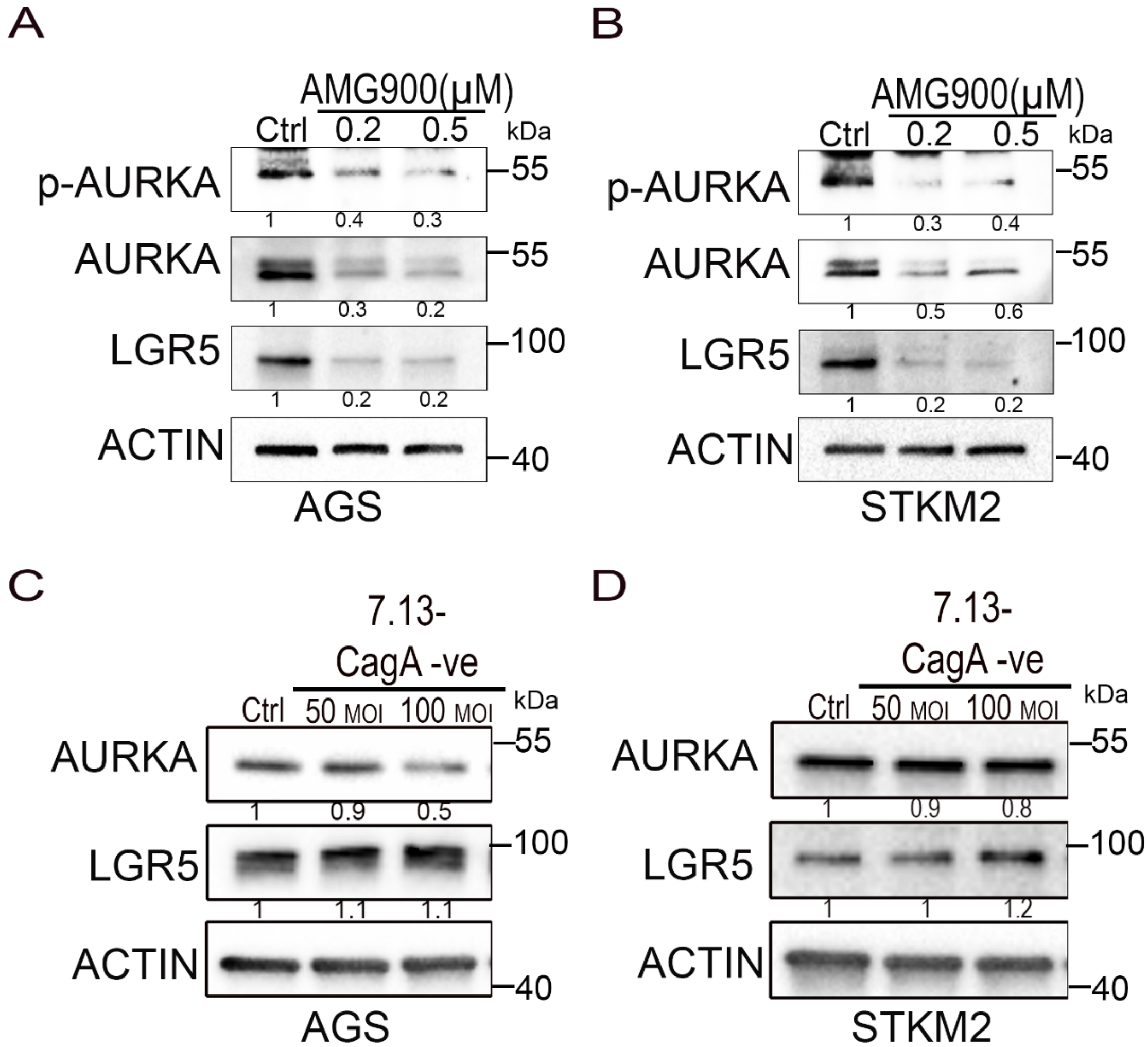

(A and B) Western blot analysis of AURKA and LGR5 in AGS and STKM2 cells with or without inhibition of AURKA with AMG-900. All data represented are representative of 3 independent experiments.

(C and D) Western blot analysis of, AURKA, and LGR5 in AGS and STKM2 cells following CagA negative (-ve) *H. pylori* infection (7.13). All data represented are representative of 3 independent experiments.

Supplementary figure 2

Supp-Figure-2

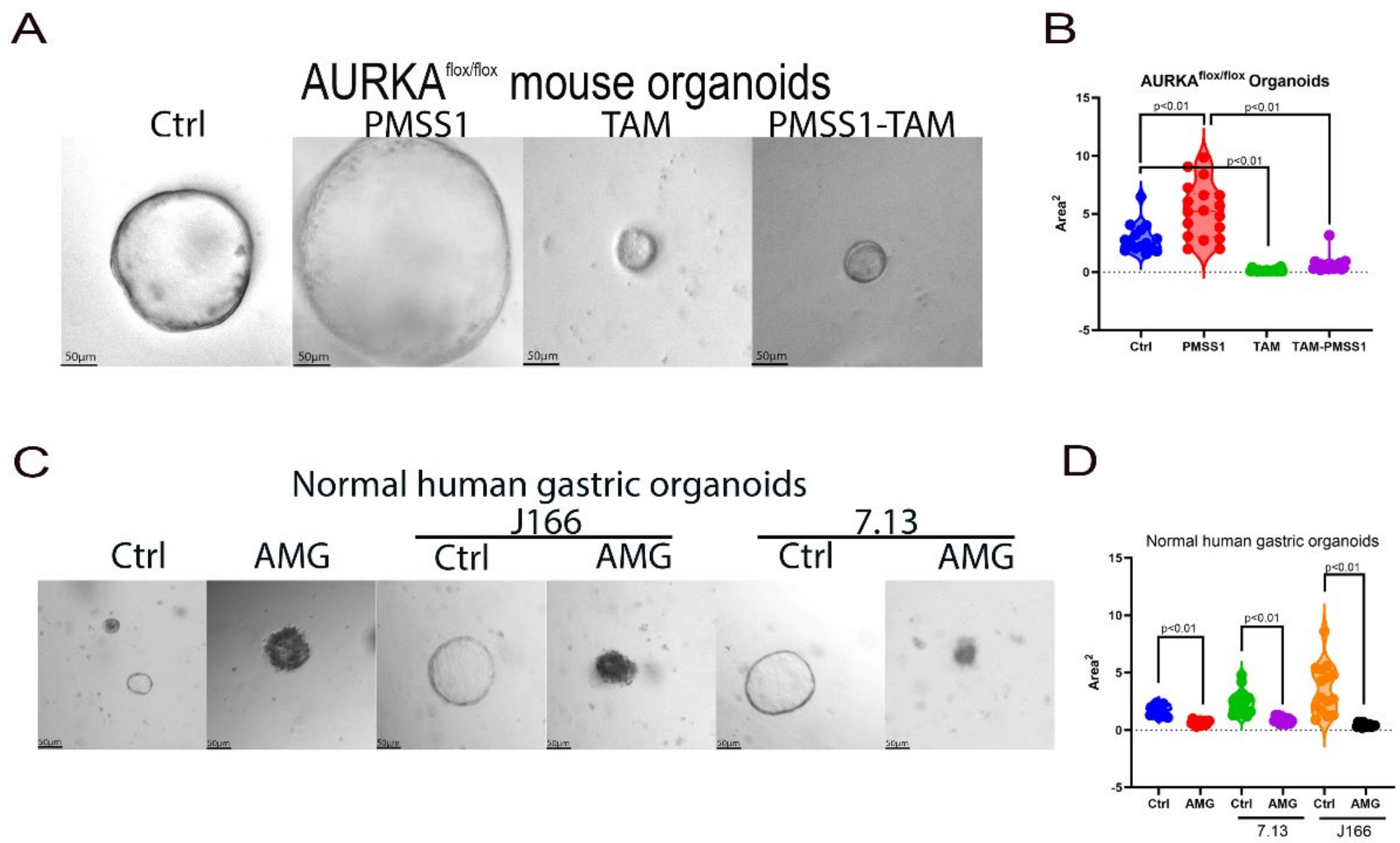

(A and B) Gastric organoids derived from *Krt19*^{Cre}/*Aurka*$^{flox/flox}$ mice, which received tamoxifen (TAM) or not (Ctrl) *in vivo*, with or without infection with *H. pylori* (PMSS1). Representative images are shown (scale bars, 50 μm) and size quantified as the mean ± SEM of the area (μm^2) (n=5). (C&D) Gastric organoids established from normal human stomach infected with *H. pylori* (7.13) with or without inhibition of AURKA with AMG-900. Representative images are shown (scale bars, 50 μm) and size quantified as the mean ± SEM of the area (μm2) (n=4).

Supplementary figure 3

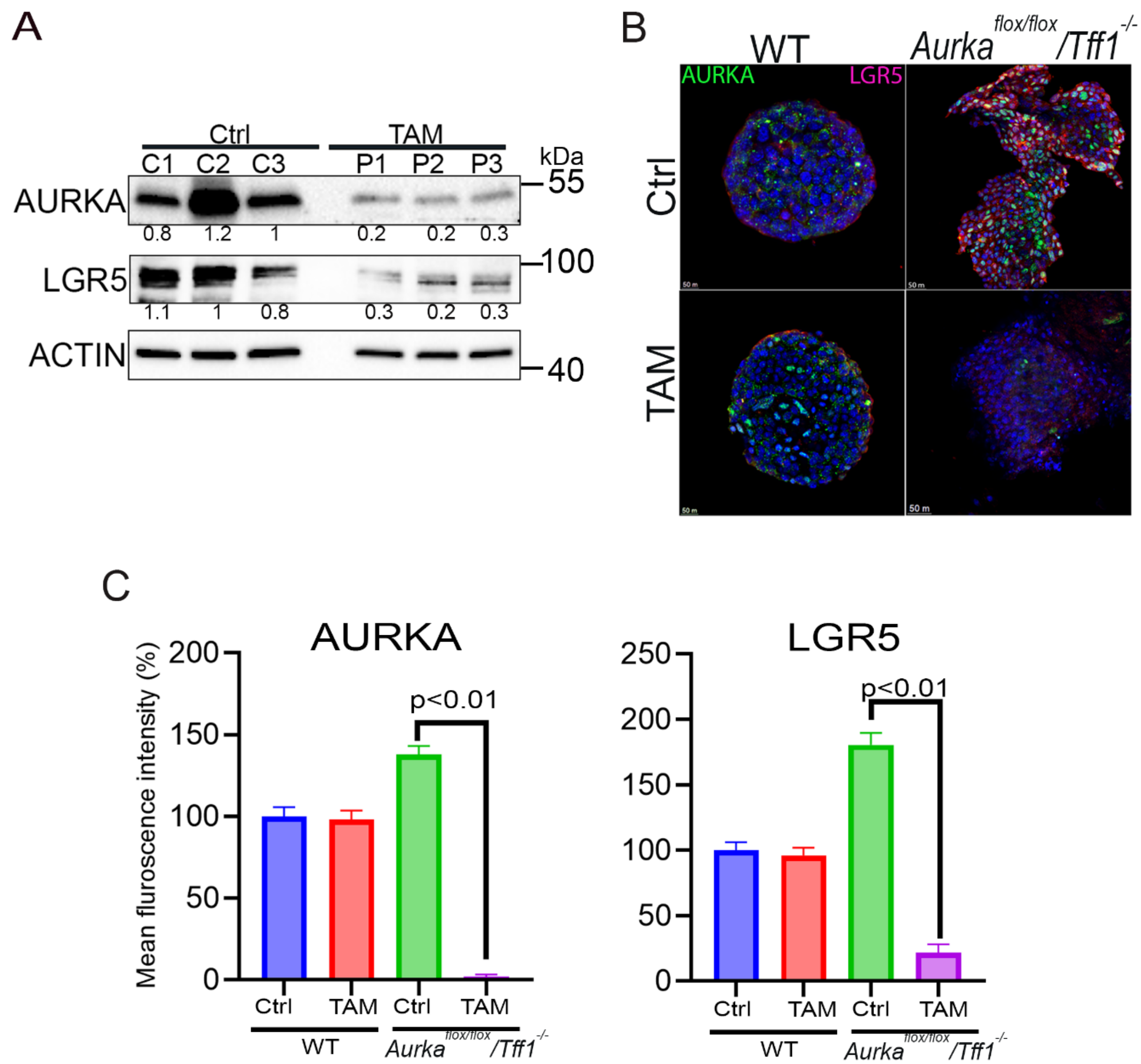

(A) Western blot analysis of AURKA and LGR5 in antral tissue of the *Krt19*^{Cre}/ *Aurka*$^{flox/flox}$*Tff1*$^{-/-}$ mice that received tamoxifen (TAM) or not (Ctrl) *in vivo (n=6)*.

(B&C) Representative image of immunofluorescence staining of AURKA and LGR5 in organoids derived from *Krt19*^{Cre}/ *Aurka*$^{flox/flox}$*Tff1*$^{-/-}$ mice that received tamoxifen (TAM) or not (Ctrl) *in vitro* (n=4).

Supplementary figure 4

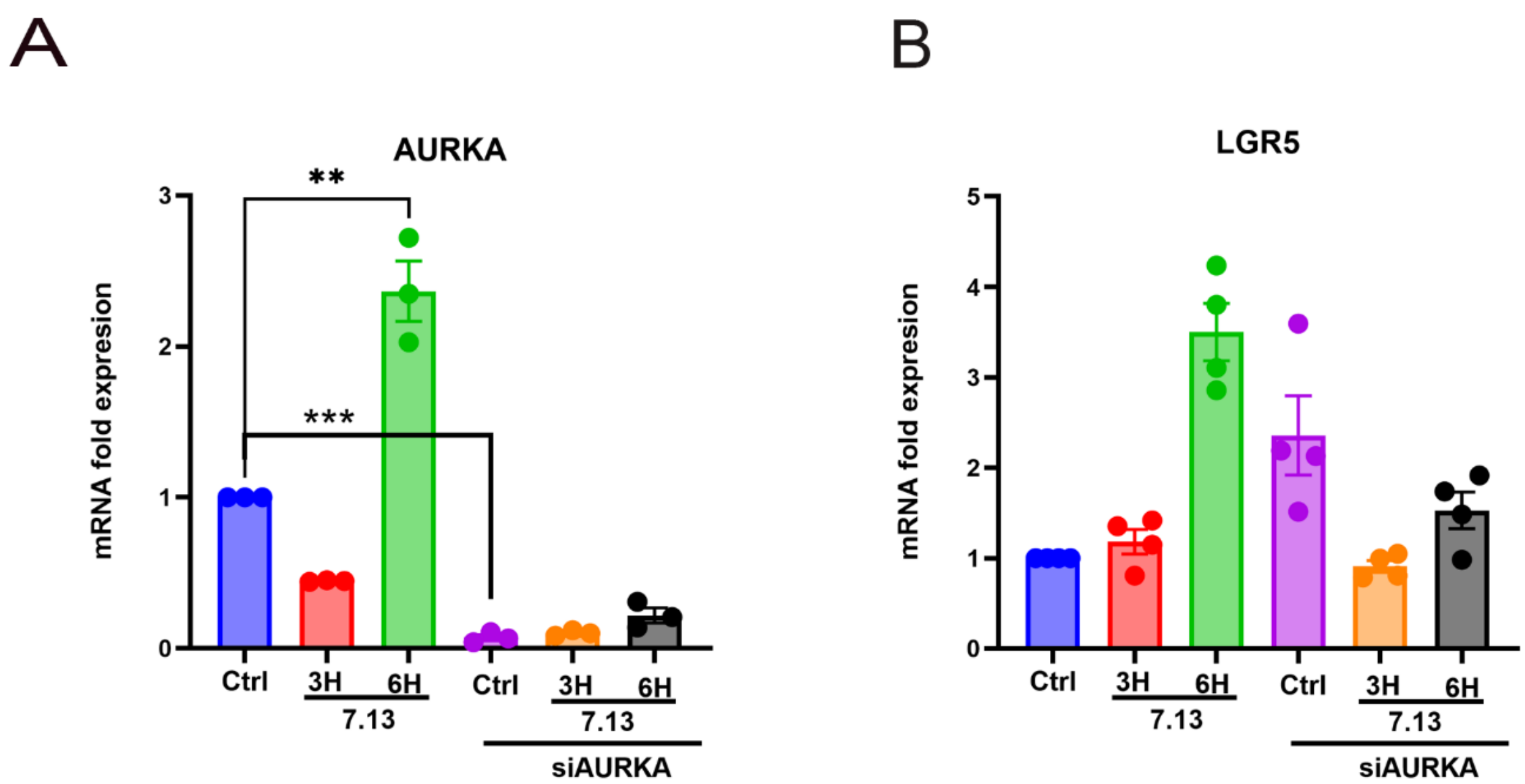

(A and B) qRT-PCR analysis of *AURKA* and *LGR5* mRNA in STKM2 cells infected with *H. pylori* (7.13) with or without knockdown of *AURKA*. All data represented are representative of 3 independent experiments.

Supplementary figure 5

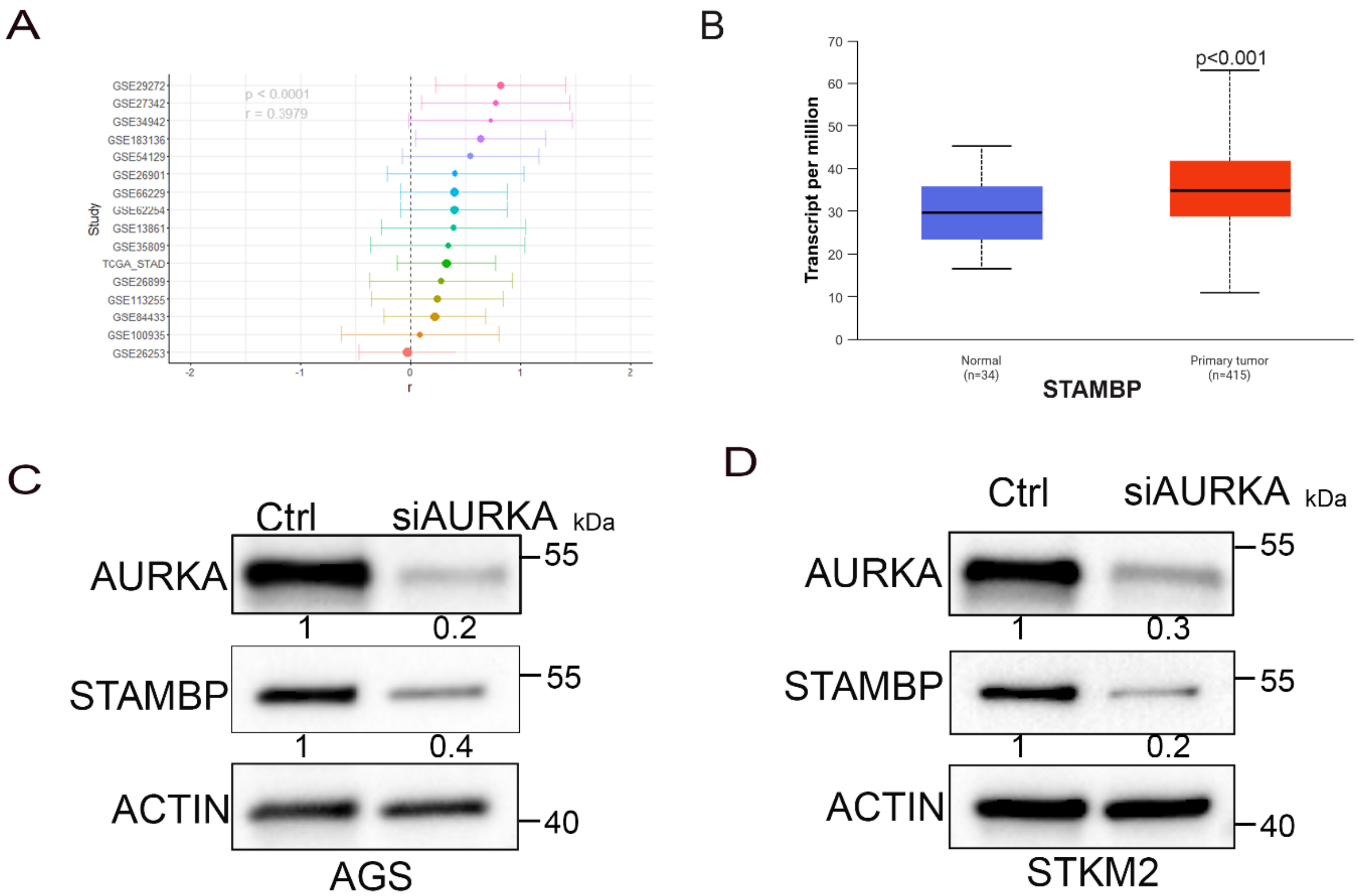

(A) Pearson's correlation test comparing *AURKA* and *STAMBP* expressions in gastric cancer GEO datasets. (B) Expression of *STAMBP* in normal human gastric tissue *versus* stomach adenocarcinoma. (C and D) Western blot analysis of AURKA and STAMBP in AGS and STKM2 cells with or without AURKA knockdown. All data represented are representative of 3 independent experiments.

Supplementary figure 6

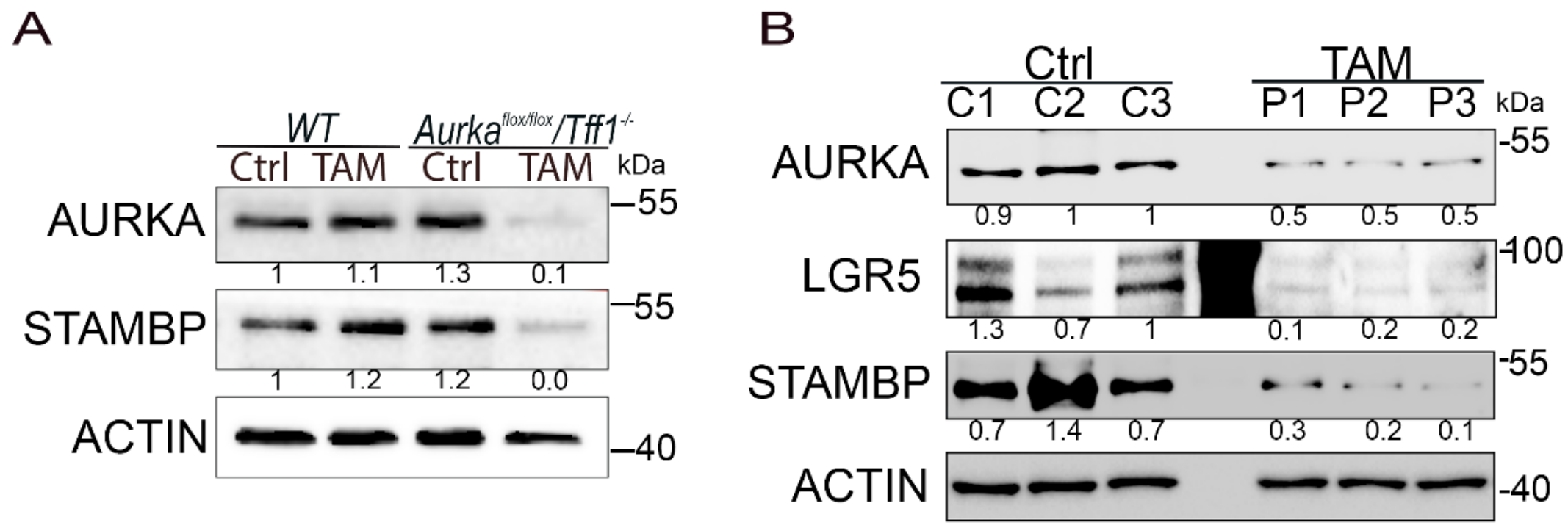

(A) Western blot analysis of AURKA and STAMBP in organoids derived from antral tissue of $Krt19^{Cre}/Aurka^{flox/flox}Tff1^{-/-}$ mice that received tamoxifen (TAM) or not (Ctrl) *in vitro*. (B) Western blot analysis of AURKA, LGR5, and STAMBP in the antral tissue of the $Krt19^{Cre}/Aurka^{flox/flox}Tff1^{-/-}$ mice that received tamoxifen (TAM) or not (Ctrl) *in vivo*. All data represented are representative of 3 independent experiments.

Supplementary figure 7

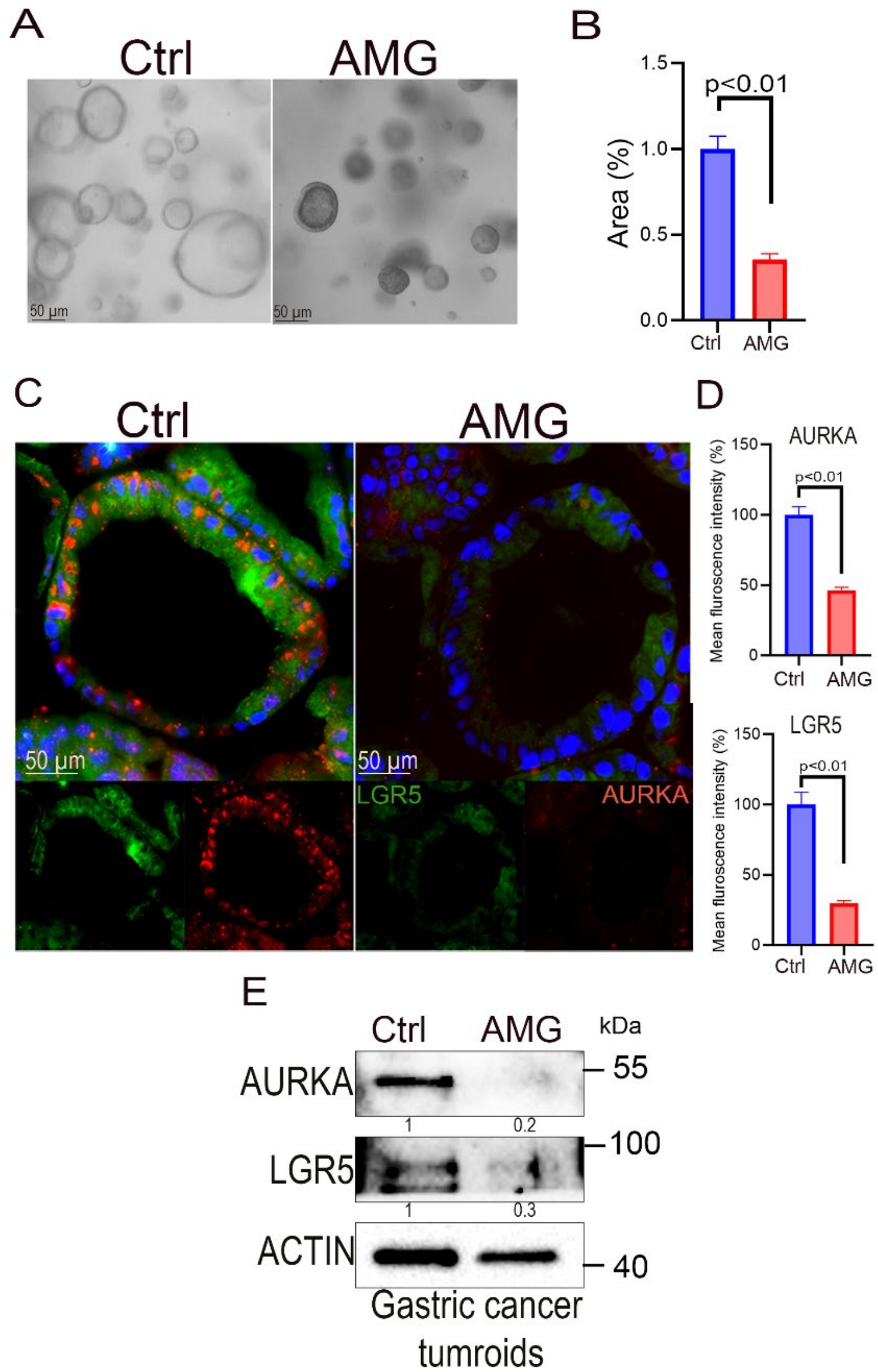

(A and B) Human gastric tumoroids were treated with or without AMG-900. Representative images are shown (scale bars, 50 μm). Size was quantified as the mean ± SEM of the area (μm^2) (n=6). (C&D) Representative immunofluorescence staining of AURKA (red) and LGR5 (green) in human gastric tumoroids treated with or without AMG-900 (scale bars, 100 μm) (n=6). (E) Western blot analysis of AURKA and LGR5 expression in human gastric tumoroids treated with or without AMG-900. All data represented are representative of 3 independent experiments.

Supplementary figure 8

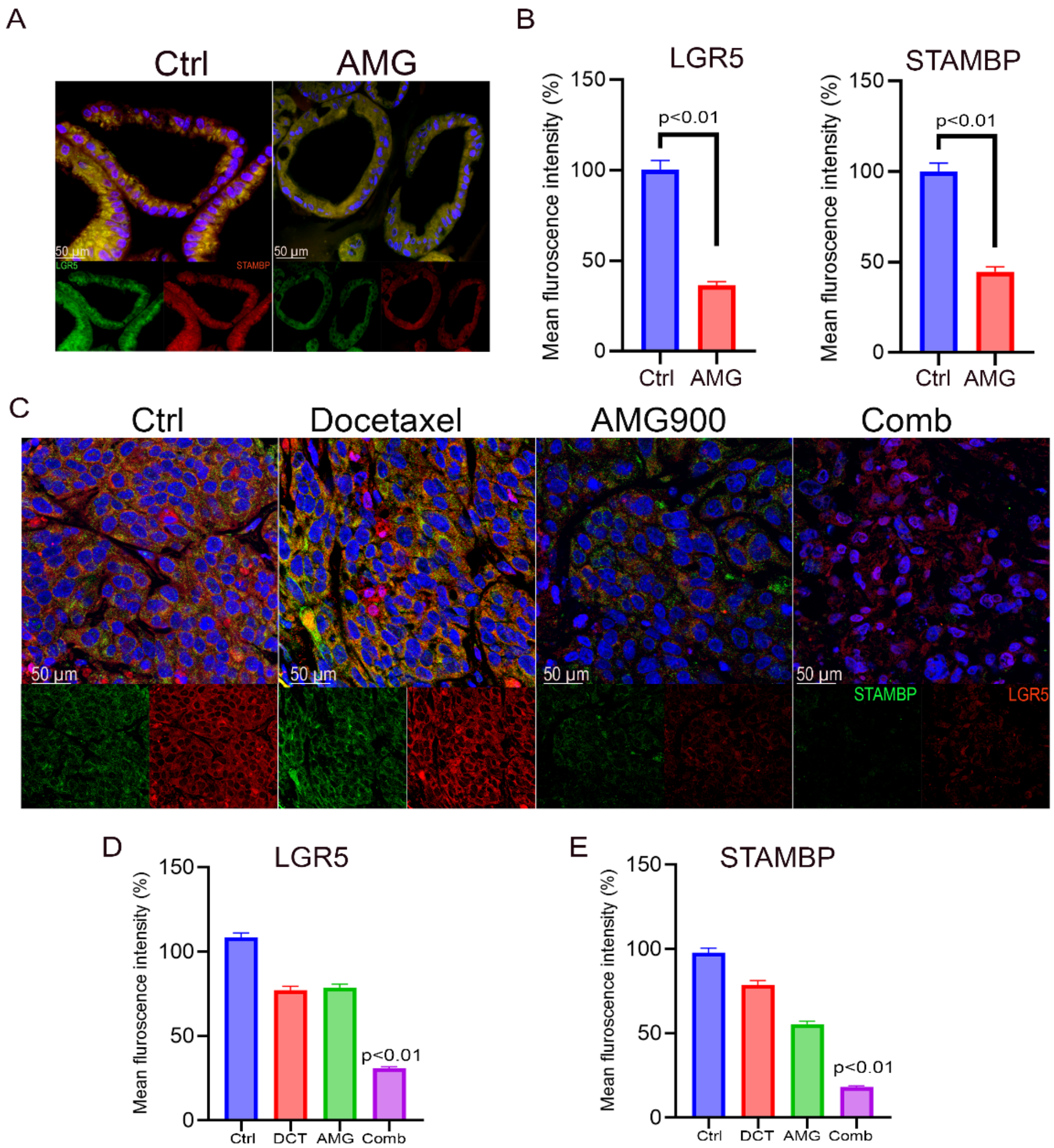

(A &B)Representative immunofluorescence staining of STAMBP (red) and LGR5 (green) in human gastric tumoroids treated with or without AMG-900 (scale bars, 100 μm) (n=6). (C,D&E) Representative immunofluorescence staining of STAMBP (green) and LGR5 (red) in PDXs treated with or without Docetaxel, AMG-900, or a combination treatment (scale bars, 100 μm). All data represented are representative of 3 independent experiments.
