# Supplementary material for: AURKA regulates LGR5 in response to Helicobacter Pylori infection by modulating its deubiquitination
Source: Cell Death Differ. Author manuscript; Available in PMC 2026 Oct 1. (PMC13628944; doi:10.1038/s41418-026-01810-w)
Supplement: Supplementary file 2 [file NIHMS2210525-supplement-Supplementary_Tables.pdf]

Table -1 Primers & Silencers

human *AURKA*	forward, 5′-GCTGGAGAGCTTAAAATTGCA-3′<br>reverse, 5′-TTTGTAGGTCTCTTGGTATGTG-3′;
human *LGR5*:	forward, 5′-CACCTCCTACCTAGACCTCAGT-3′<br>reverse, 5′-CGCAAGACGTAACTCCTCCAG-3′;
human *HPRT1*	forward, 5′-ACCCTTTCCAAATCCTCAGC-3′<br>reverse, 5′-GTTATGGCGACCCGCAG-3′;
mouse *Aurka*	forward, 5′- CTGGATGCTGCAAACGGATAG-3′<br>reverse, 5′- CGAAGGGAACAGTGGTCTTAACA-3′;
mouse *Lgr5*	forward, 5′-CAGCCTCAAAGTGCTTATGCTG-3′<br>reverse, 5′- TGGCACGTAACTGATGTGG -3
mouse *Hprt*	forward, 5′- CCTAAGATGAGCGCAAGTTGAA-3′<br>reverse, 5′- CCACAGGACTAGAACACCTGCTAA-3′.
Silencer™ Select Negative Control siRNA	Thermofisher Scientific # 4390843
Silencer™ Pre-Designed AURKA siRNA	ThermoFisher Scientific (ID:139201 and ID: 139203) # AM16708
Silencer™ Pre-Designed STAMBP siRNA	ThermoFisher Scientific (ID: 17954 and ID: 18048) # AM16708

Table -2 Antibodies

AURKA	Cell Signaling (D3E4Q)Rabbit mAb #14475
phospho-AURKA (Thr288)	Cell Signaling (C39D8)Rabbit mAb #3079
SOX9	Cell Signaling (D8G8H)Rabbit mAb #82630
STAMBP	Cell Signaling (5245) Rabbit mAb #5245
Ubiquitin	Cell Signaling (E6K4Y) XP® Rabbit mAb #20326
LGR5	Thermofisher Rabbit Polyclonal Antibody # PA5-23000
Mouse Lgr5/GPR49	R&D Mouse Lgr5/GPR49 Alexa Fluor® 488-conjugated Antibody # FAB8240G
Anti-Mouse Aurka	RayBiotech Rabbit Anti-Mouse Aurka (N-term) Antibody #: 102-21947
CagA	AUSTRAL Biologicals Rabbit anti-Helicobacter pylori Cag antigen IgG fraction (polyclonal) # HPP-5003-9
Actin	Millipore-sigma Monoclonal Anti-β-Actin Clone AC-1 #5A5441
AMG-900	NedChemExpress #HY-13253
MG132	NedChemExpress # HY-13259
Isotype normal mouse IgG	Santa Cruz Biotechnology Inc. # sc-2025
Dynabead Protein G	ThermoFisher Scientific # 10003D

	Label	Number	Antral Thickness	Diagnosis	Antr_Chron Infl	Antr_Acu Infl	Antr_Dysplasia	Antral hyperplasia	Antral Atrophy	Fundus gastritis	Fundus Metaplasia
No TAM	AF	158	1262	**Invasive Adenocarcinoma**	3	1	3	3	0	1	0
	AF	159	1156	Intramucosal CA in an adenoma	3	3	2.5	3	0	0	0
	AF	160	921	Intramucosal CA in an adenoma	2	1.5	2.5	3	0	0	0
	AF	161	837	Intramucosal CA in an adenoma	1.5	2.5	2.5	3	0	0	0
	AF	162	844	Intramucosal CA in an adenoma	3	1.5	2.5	3	0	2	0
	AF	70	1027	Low Grade Antral Adenoma	2.5	0.5	1	3	0	0	0
	AF	71	825	Intramucosal CA in an adenoma	3	2	2.5	3	0	1	0
	AF	72	639	Low Grade Antral Adenoma	1	0	1	2	0	2.5	2
	AF	73	473	Low Grade Antral Adenoma	1	0	1	2	0	1	2

TA M	AF	58	404	Low Grade Antral Adenoma	1.5	0	1	2	0	1	0
	AF	59	463	Low Grade Antral Adenoma	2	2	1	2	0	0	0
	AF	60	574	Invasive Adenocarcinoma	2	2	3	2	0	1	0
	AF	61	721	Low Grade Antral Adenoma	3	0	1	2	0	0	0
	AF	62	615	High Grade Antral Adenoma	1	0	2	2	0	0	0
	AF	63	588	High Grade Antral Adenoma	2.5	0	2	2	0	0	0
	AF	64	689	Intramucosal CA in an adenoma	3	3	2	2	0	0	0
	AF	65	662	High Grade Antral Adenoma	1.5	0	2	2	0	0	0
